# Germplasm Screening Using DNA Markers and Genome-Wide Association Study for the Identification of Powdery Mildew Resistance Loci in Tomato

**DOI:** 10.3390/ijms232113610

**Published:** 2022-11-06

**Authors:** Jiyeon Park, Siyoung Lee, Yunseo Choi, Girim Park, Seoyeon Park, Byoungil Je, Younghoon Park

**Affiliations:** 1 Department of Horticultural Bioscience, Pusan National University, Miryang 50463, Korea; 2 Life and Industry Convergence Research Institute, Pusan National University, Miryang 50463, Korea

**Keywords:** powdery mildew, tomato, genome wide association study, quantitative trait loci, germplasm screening

## Abstract

Powdery mildew (PM), caused by *Oidium* spp. in tomato, is a global concern that leads to diminished yield. We aimed to evaluate previously reported DNA markers linked to powdery mildew resistance (PMR) and identify novel quantitative trait loci (QTLs) for PMR through a genome-wide association study in tomato. Sequencing analysis of the internal transcribed spacer (ITS) of a PM strain (PNU_PM) isolated from Miryang, Gyeongnam, led to its identification as *Oidium neolycopersici*. Thereafter, a PM bioassay was conducted for a total of 295 tomato accessions, among which 24 accessions (4 *S. lycopersicum* accessions and 20 accessions of seven wild species) showed high levels of resistance to PNU_PM. Subsequently, we genotyped 11 markers previously linked to PMR in 56 accessions. PMR-specific banding patterns were detected in 15/22 PMR accessions, while no such bands were observed in the powdery mildew-susceptible accessions. The genome-wide association study was performed using TASSEL and GAPIT, based on the phenotypic data of 290 accessions and 11,912 single nucleotide polymorphisms (SNPs) obtained from the Axiom^®^ Tomato SNP Chip Array. Nine significant SNPs in chromosomes 1, 4, 6, 8, and 12, were selected and five novel QTL regions distinct from previously known PMR-QTL regions were identified. Of these QTL regions, three putative candidate genes for PMR were selected from chromosomes 4 and 8, including two nucleotide binding site-leucine rich repeat class genes and a receptor-like kinase gene, all of which have been identified previously as causative genes for PMR in several crop species. The SNPs discovered in these genes provide useful information for understanding the molecular basis of PMR and developing DNA markers for marker-assisted selection of PMR in tomato.

## 1. Introduction

Powdery mildew (PM) in tomatoes *(Solanum lycopersicum* L., 2n = 2x = 24) is a common concern worldwide, especially in areas with cool and dry climates. It reduces production by more than 50%, depending on the growth stage of the infected plants and the surrounding environment. The major pathogens involved in PM are *Oidium neolycopersici*, *Oidium lycopersici*, and *Leveillula taurica*, which belong to Ascomycota [1]. Among them, *O*. *neolycopersici* is the main cause of PM in tomato plant cultivation. When infected, a small circular, opaque white powdery sign appears on the upper surface of the lower leaf of the plant, which then spreads rapidly to cover the entire leaf [2]. When the symptoms become severe, the leaves fall off prematurely, leading to yellowing and necrosis, which ultimately interferes with photosynthesis, thereby lowering the growth rate and fruit quality of crops [3].

For economical, sustainable, and eco-friendly cultivation, there is a need for use of PM-resistant varieties. However, commercial varieties known to be PM-resistant have hardly been developed, in addition to which, only some related wild species showing potential for use as breeding materials have been discovered [4,5]. To date, the PM resistance (PMR) genes identified in wild tomato species include five single dominant genes (*Ol-1*, *Ol-3*, *Ol-4*, *Ol-5*, and *Ol-6*), one single recessive gene (*ol-2*), and three quantitative trait loci (QTLs; *Ol-qtl1*, *Ol-qtl2*, and *Ol-qtl3*) [5]. Among them, *Ol-4* (derived from *S*. p*eruvianum* accession LA2172) and *Ol-6* (derived from a breeding line of unknown origin) were mapped very closely on the short arm of chromosome 6 and reported as different alleles at the same locus (*Ol-1*,*3*) [6]. In addition, an allelic relationship of the same gene was proposed between *Ol-1* (*S*. *habrochaites* accession G1.1560) and *Ol-3* (*S*. *habrochaites* accession G1.1290), which is located on the long arm of chromosome 6 [6]. The *Ol-5* derived from *S*. *habrochaites* accession PI247087 was mapped in the region adjacent to *Ol-1*,*3*, and the three QTLs, *Ol-qtl1*, *Ol-qtl2*, and *Ol-qtl3* derived from *S*. *neorickii* accession G1.1601 were mapped to the proximal region of *Ol-5* on chromosome 6 and the short and long arms of chromosome 12, respectively [6,7,8]. In particular, *Ol-qtl2* is associated with the resistance locus (*Lv*) of *Leveillula taurica*, another pathogen of PM [7]. The recessive gene *ol-2* found in *S*. *lycopersicum* var. *cerasiforme* accession LA1230 is located on chromosome 4 and is the only PMR gene (*Slmlo1*) in tomato that has been cloned so far [9,10,11].

Molecular markers reported for tomato PMR are known as gene-linked markers, except for the *ol-2* gene-based marker. The use of gene-linked markers in marker-assisted selection (MAS) may result in a reduction in the selection accuracy, due to recombination between the marker and gene at the time of germ cell meiosis. Therefore, high-resolution mapping aimed at the development of gene-based markers is of immense importance. Traditional genetic mapping and QTL analysis use a bi-parental population derived from a cross between two parental lines, but the process of creating a progeny is time-consuming and the detection of QTLs gets limited to those already existing in the parental lines. On the other hand, a genome-wide association study (GWAS) does not require the creation of a segregation population, because it considers genetic variations in natural populations with various genetic bases, and in addition, allows for high-resolution mapping due to the high frequency of recombination between the loci [12]. GWAS mainly uses information on single nucleotide polymorphisms (SNPs), which are the most common and evenly occurring mutations in the entire genome, as genotype data [13]. The development of next-generation sequencing (NGS) technology has facilitated whole genome sequencing and re-sequencing within a shorter period of time, as compared to that taken upon doing the same using conventional Sanger sequencing, and thereby, accelerated the discovery of all genetic mutations. However, with an increase in the number of samples to be analyzed, there is considerable cost and effort involved. In order to compensate for this, genotyping by sequencing and SNP microarray chip technologies are mainly used as high-throughput genotyping methods for some SNPs in the whole genome. Genotyping by sequencing (GBS) is based on next-generation sequencing technology and reads only a short sequence around the recognition sites of a specific restriction enzyme, thereby reducing the cost and enabling rapid discovery of SNPs. However, in GBS, there is a limitation that the SNP to be searched for may vary depending on the target sample, and the quality of the SNP may be low due to the difference in read depth for each nucleotide sequence [14]. On the other hand, the SNP chip array attaches a high-density probe to detect tens of thousands to millions of SNPs on the semiconductor surface, so that consistent SNP information in the whole genome can be quickly and accurately obtained from a large number of samples [15,16]. Linkage mapping for genes involved in target traits and GWAS using SNP chip arrays for new QTLs have been performed in cabbage, peanuts, and wheat [17,18,19].

Of the SNP chip arrays produced so far in tomato, two are publicly available, including SolCAP Tomato 2013 (Illumina Infinium^®^, 9K) [20] and Axiom^®^ Tomato Genotyping Array (Affymetrix Axiom^®^, 52K) [21]. SolCAP Tomato 2013 is based on the transcriptome information from cultivated tomatoes, and used for the gene-based mapping of metabolite content in tomato [22,23], while Axiom^®^ Tomato Genotyping Array is based on the whole genome resequencing of commercial F1 hybrid cultivars and used for QTL detection for yield, fruit quality, and abiotic stress [21]. To the best of our knowledge, however, no GWAS for PMR in tomato has been reported to date.

In order to develop PM-resistant tomato varieties, it is necessary to continuously discover breeding materials with high PMR. It is also necessary to identify PMR genes and molecular markers that can be selected by application of various analysis techniques that use not only bi-parental or multiparent segregation populations, but also natural populations. Therefore, this study aimed to (1) screen PMR from a population of diverse tomato genetic resources, including wild species, (2) search for PMR loci based on previously reported PMR-associated markers, and (3) identify novel PMR-associated QTL(s) and candidate genes through SNP genotyping and GWAS, based on the Axiom^®^ Tomato Genotyping Array.

## 2. Results

### 2.1. Pathogen Identification

**Figure 1:**
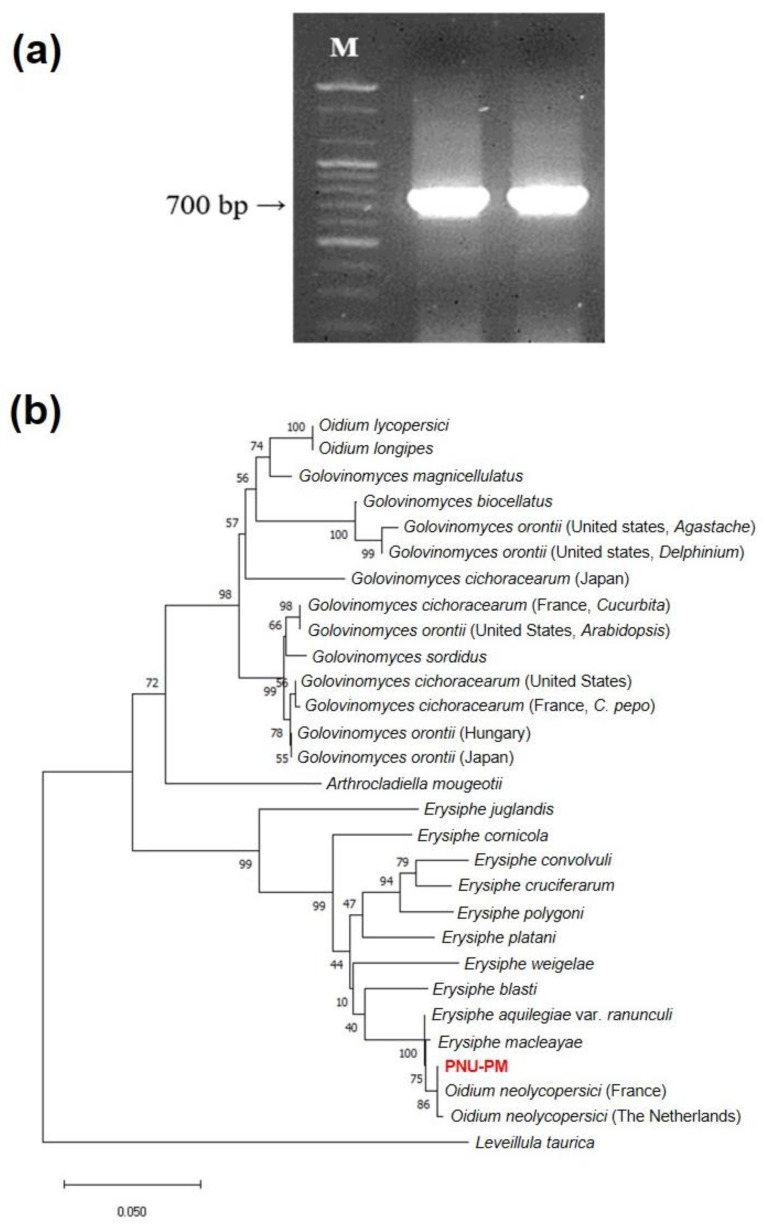
(**a**) Polymerase chain reaction (PCR) amplification of the internal transcribed spacer (ITS) region of the *Oidium neolycopersici* isolate ‘PNU-PM’ using the PMITS1 and PMITS2 primers [24]; (**b**) A phylogenetic tree of powdery mildew pathogens constructed using the neighbor-joining method based on the ITS sequences of ribosomal DNA. The duplicate PCR amplicons were loaded on an agarose gel, and the specificity of the ITS region of ‘PNU-PM’ was confirmed based on the size (700 bp) of the PCR amplicons. Lane ‘M’, 100 bp DNA size marker. The ITS of the ‘PNU-PM’ powdery mildew pathogen (red in **b**) isolated in this study was found to be identical to that of *O. neolycopersici*.

We sequenced the internal transcribed spacer (ITS) region of the PM pathogen (named PNU-PM) found on the leaves of the tomato plants growing in the greenhouse of Pusan National University’s Miryang Campus, Korea. The length of the ITS sequence of PNU-PM amplified using PMITS1 and PMITS2 primers was found to be about 700 bp (Figure 1a). The ITS sequences for a total of 29 fungal species including PNU-PM, *L. taurica*, and pathogens used in Kiss et al. [24] were aligned using Clustal X, to create a phylogenetic tree.

The ITS sequence of PNU-PM (NCBI accession number: MW082786.1) matched 100% with that of the *O*. *neolycopersici* France isolate and 99.8% with that of the Netherland isolate and was most closely tied with a bootstrap value of 86% on the phylogenetic tree (Figure 1b). The ITS sequences of *O*. *lycopersici* and *L*. *taurica*, the other PM-causing pathogens in tomatoes, showed a 79.5% and 72.6% match, respectively, with that of PNU-PM, but were distinct from that of *O*. *neolycopersici* (Figure 1b). In addition, BLASTn search of the ITS sequence of PNU-PM showed a 100% match (query cover = 100% and E-value = 0) with the ITS sequence of 42 *O. neolycopersici* isolates.

### 2.2. PM Bioassay

**Figure 2:**
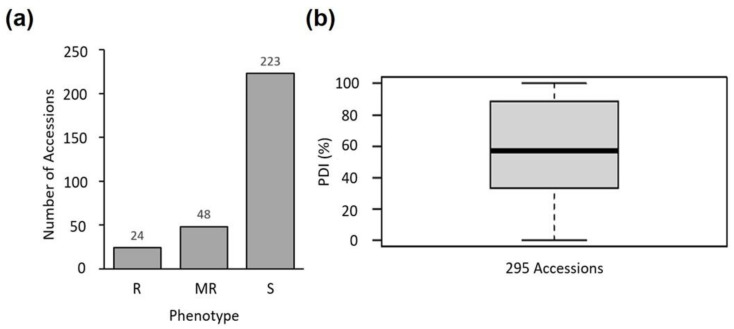
(**a**) Phenotypic distribution of resistance to powdery mildew caused in tomato by *Oidium neolycopersici*; (**b**) A box plot showing the statistics of distribution. In the box plot, the bold line in the middle of the gray box represents the median (cumulative percentage 50% = 57.14%), while the upper and lower edges of the box represent the cumulative percentage 75% = 88.89% and 25% = 33.33%, respectively.

The PM bioassay for 295 accessions (244 cultivars and 51 wild species accessions) including six PM-resistant and PM-susceptible (PMS) controls, revealed a total of 24 accessions that were highly resistant to PM [R, percent disease intensity (PDI) < 10%] (Table S1 (see Supplementary Materials) and Figure 2). Most of the highly resistant accessions were wild species, of which no symptoms (PDI = 0) were observed in 17 accessions (*S. galapagense* accession LA1141, *S. pennellii* accessions LA1674 and LA1809, *S. cornerliomulleri* accession LA1274, *S. chilense* accessions LA1963 and LA2932, *S. pimpinellifolium* accession LA2181, *S. chmielewskii* accession LA1274, *S. peruvianum* accession LA2744, *S. habrochaites* accession PI126445, and *S. lycopersicum accessions* KNU17, A1161, A1162, and A1216), including the *S. chilense* accessions LA0458 and LA1969 that are resistant to *L. taurica* [5] (Table S1).

A total of 48 accessions were moderately resistant (MR, PDI = 10–30%), including LA2172 (*S*. *peruvianum*), which carries the resistance gene *Ol-4*. The remaining 223 accessions were susceptible (S, PDI > 30%), among which 204 lines were *S. lycopersicum*, including susceptibility controls, PNU-PMS (*S*. *lycopersicum*) and Moneymaker as well as one *S. habrochaites* accession (PI247087) carrying the resistance gene *Ol-5* (Table S1).

### 2.3. Validation of the PMR-Linked Markers

Eleven previously reported PMR-linked DNA markers were genotyped in a total of 56 accessions, including 22 accessions that showed resistance (R, PDI < 10%), 28 accessions that showed sensitivity (S, PDI = 100%), and 6 controls (Table 1). PMR-specific marker genotypes were observed in 15 of the resistant accessions but in none of the susceptible accessions. In the case of the recessive resistance gene *Slmlo1* at the locus *ol-2*, for which a gene-based marker has been developed, there was no accession carrying the marker genotype for the *Slmlo1* allele, but the marker genotype specific to *Slmlo1.1*, another allele of the gene, was observed in the resistant accessions KNU16 and KNU17 (Table 1).

In the case of the PMR QTLs, *Ol-qtl1* was observed in five wild accessions [LA0716, LA1674, LA1809 (*S*. *pennellii*), LA1274 (*S*. *cornerliomulleri*), and LA2663 (*S*. c*hmielewskii*)] and LA1969 (*S*. *chilense*) carrying the *Lv* gene. *Ol-qtl3* was found in 10 wild accessions [LA0716, LA1340, LA1674, and LA1809 (*S*. *pennellii*), LA1274 (*S*. *cornerliomulleri*), LA2663 (*S*. *chmielewskii*), LA2744 (*S*. *peruvianum*), and PI126445 and PI308182 (*S*. *habrochaites*)] including the *Ol-4*-derived accession LA2174 (*S*. *peruvianum*). The accessions that displayed PMR in the bioassay but did not present a PMR-specific marker genotype included LA1141 (*S*. *galapagense*), LA1272 (*S*. *pennellii*), LA1963 and LA2932 (*S*. *chilense*), LA2181 and LA2184 (*S*. *pimpinellifolium*), A1161, A1162, A1164, and A1216 (*S*. *lycopersicum*), and LA0458 (*S*. *chilense*), an accession carrying the *Lv* genes.

### 2.4. Quality Evaluation of the Raw SNP Genotyping Data

A total of 290 accessions were genotyped using the Axiom^®^ Tomato Genotyping Array, resulting in the raw data for 51,214 SNPs. Following that, for SNP quality evaluation in the wild species, Dish QC (DQC), call rate, and heterozygosity rate were analyzed for 182 accessions, excluding the 160 cultivated tomato accessions (Table S2). The average DQC of the *S*. *lycopersicum* accession was as high as 0.79–0.99, while that of the wild species accessions was very low (0.04–0.15). In the wild species, the DQCs of *S. cheesmaniae*, *S. galapagense*, and *S. pimpinellifolium*, which are known to exhibit high crossing compatibility with *S. lycopersicum* [25], were 0.15, 0.14, and 0.10, respectively, while those of other wild species were in a lower range of 0.03–0.04 (Table 2).

The heterozygosity rate was as low as 5.43–7.44% in the cultivated species, but as high as 7.74–33.44% in the wild species. For call rate, the minimum was 88.64% (SAL1875, *S. pimpinellifolium*), and the maximum was 98.85% (LA3871, *S*. *lycopersicum*), thereby indicating that the overall SNP chip array genotyping was performed properly; however; a relatively low SNP call rate in the wild species implied higher number of missing call signals in this set as compared to that in the cultivated species (Table S2). A correlation analysis between the three factors of DQC, call rate, and heterozygosity rate revealed a significant correlation coefficient (0.672, *p* < 0.01) of DQC and call rate, which indicated that the higher the DQC, the higher the call rate. However, the correlation coefficients between DQC and heterozygosity rate as well as call rate and heterozygosity rate were –0.807 and –0.881, respectively, indicating a strong negative correlation between these factors, i.e., the heterozygosity rate tended to be high when the DQC or call rate was low.

### 2.5. Homology Analysis of the SNP-Flanking Probe Sequence among Species

To identify the cause of low DQC, call rate, and high heterozygosity rate in the wild species, sequence homology analysis was performed between the probe sequence of 51,214 SNP loci used in the Axiom^®^ Tomato Genotyping Array and the reference genome assembly of 5 cultivated and wild species obtained from National Center for Biotechnology Information (NCBI). Upon alignment of the probe sequences on the reference genome of each species, all of the probe sequences of the 51,214 SNP loci were mapped in *S. lycopersicum*, while only 36,811 SNP loci (71.88% of the total) were mapped in the wild species *S. chilense*, 38,335 SNP loci (74.85%) in *S. habrochaites*, 38,117 SNP loci (74.43%) in *S. pennellii*, 49,738 SNP loci (97.12%) in *S. pimpinellifolium*, and 42,550 SNP loci (83.08%) in *S. arcanum*; thus, as compared to *S. lycopersicum*, there was low homology with the SNP-flanking probe sequence used in the Axiom^®^ Tomato genotyping array in the wild species.

**Figure 3:**
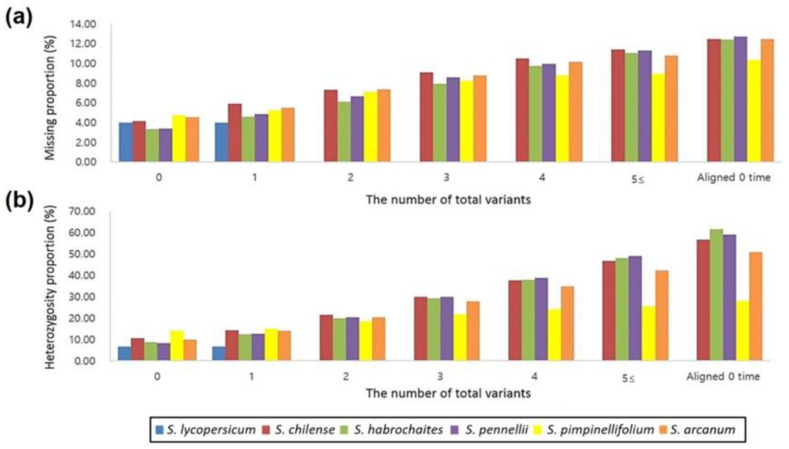
(**a**) The relationship between the proportion of failed (missing) or heterozygous; (**b**) SNP genotype data and the homology of the Axiom^®^ Tomato Genotyping Array probe sequences and reference genome sequences in different tomato species. The probe sequences for the 51,214 SNP loci were compared to the reference genome assemblies, to calculate the number of sequence variants. Aligned 0 time on the horizontal axis indicates the condition under which the probe sequence could not align with the reference genome assembly.

Further, we assessed the relationship of the number of nucleotide sequence variations between the reference genome and probe sequences for each species with the ratio of SNPs genotyped as missing or heterozygous on the SNP chip array (Figure 3). In the cultivar accessions, there was no or only one nucleotide sequence variation, and the proportion of SNPs that were genotyped as missing or heterozygous were 3.97–3.98% and 6.63–6.64%, respectively. In the wild species, the ratio of missing/heterozygous genotyping for SNPs with a probe sequence that perfectly matched the reference genome sequence was 10.48% in *S. chilense*, 8.66% in *S. habrochaites*, 8.27% in *S. pennellii*, 14.01% in *S*. *pimpinellifolium*, and 9.80% in *S*. *arcanum*. Whereas, among SNPs whose probe sequences did not map to the reference genome (aligned 0 times), the ratio of missing/heterozygosity was 56.61% in *S*. *chilense*, 61.64% in *S. habrochaites*, 58.96% in *S*. *pennellii*, 28.00% in *S*. *pimpinellifolium*, and 50.78% in *S*. *arcanum*. As the overall degree of nucleotide sequence variations between the reference genome and probe sequences increased, there was a proportional increase in the ratio of SNPs genotyped as missing or heterozygous.

### 2.6. SNP Validation Based on Cleaved Amplified Polymorphic Sequence (CAPS) and Derived Cleaved Amplified Polymorphic Sequence (dCAPS) Markers

To verify the reliability of the SNP chip genotyping results of wild species, six SNPs were converted into CAPS or dCAPS markers and the genotypes of the highly resistant and highly susceptible accessions were reanalyzed (Table 3). Of the 22 resistant accessions, SNP call in the SNP chip array was ‘missing’ in 3 accessions for the SNP AX-95781451, 1 for AX-95767557, 4 for AX-95784118, 3 for AX-95809776, and 2 for AX-95814666, while the SNPs were genotyped as heterozygous in 6 accessions for AX-95781451, 3 for AX-95767557, 4 for AX-95784118, 18 for AX-95773152, 8 for AX-95809776, and 10 for AX-95814666.

However, all CAPS or dCAPS markers converted from the six SNPs showed homozygous genotypes, and this trend was mainly observed in wild species accessions, except for the case of AX-95773152 in three cultivars, A1161, A1162, and A1164. In 28 susceptible lines mainly composed of cultivars, CAPS or dCAPS markers for all 6 SNPs showed a showed homozygous genotype, and the test results between the SNP chip array and the CAPS or dCAPS markers matched, except for in case of the genotype of AX-95781451 in one accession, A1345.

### 2.7. SNP Filtering for Population Structure and GWAS

Filtering SNPs under the conditions of minor allele frequency >5%, missing proportion <5%, and heterozygosity proportion <10% led to the selection of 28,374 (55.4%) SNPs. Further, to avoid low genotyping error, we filtered SNPs with a heterozygosity rate lower than 10% in the wild species, which led to a final selection of 11,912 (23.2%) SNPs (Table S3).

### 2.8. Population Structure Analysis

**Figure 4:**
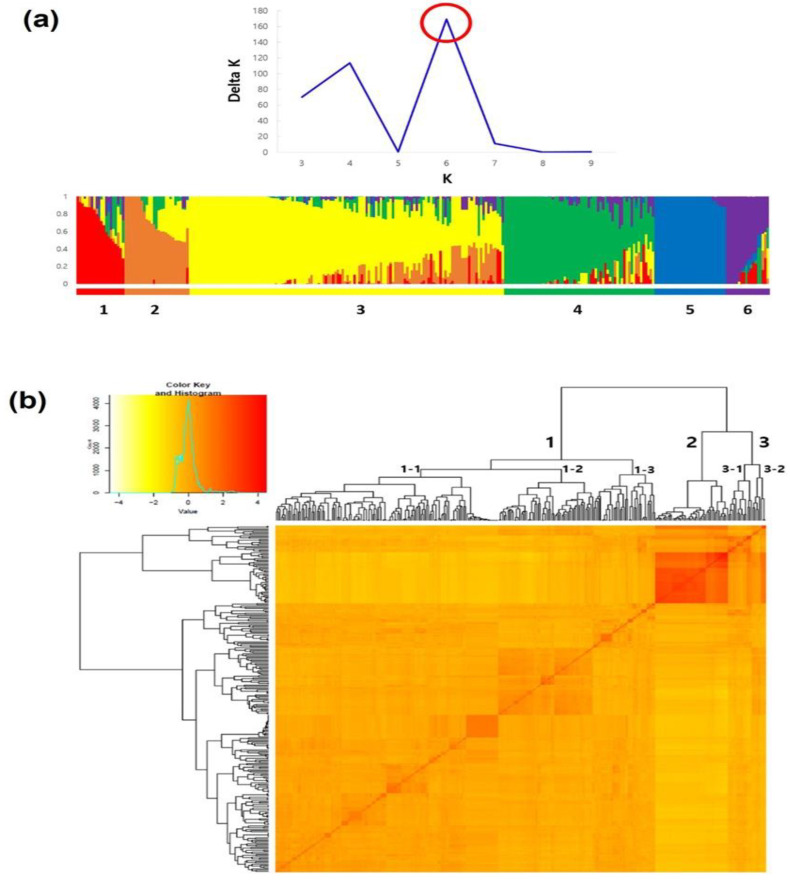
(**a**) Delta K graph calculated using the Evanno method (**top**) and optimal population structure (delta K = 6) cluster plot (**bottom**). The optimal population number has been indicated using a red circle; (**b**) Kinship heatmap plot and dendrogram for 290 accessions drawn using the genotype data of 11,912 SNPs. The genetic distance between accessions was calculated using the VanRaden algorithm.

Population structure analysis of 290 accessions was performed based on the filtered 11,912 SNPs, using the STRUCTURE analysis (Figure 4a). The optimal number of subgroups (delta K) of the group was 6. Most of the lines belonging to cultivar *S*. *lycopersicum* were divided into Groups 1, 2, 3, and 4, with 16 PGRBC lines and BT0902 lines in Group 1, 27 PGRBC lines in Group 2, and 97 PGRBC lines and *lycopersicoides* introgression in Group 3. There were 21 *lycopersicoides* IL lines, 7 *hirsutum* IL lines, and 7 other lineages. Group 4 included 9 accessions of PGRBC, 1 accession of *lycopersicoides* IL (LA3875), and 16 other accessions.

Cherry tomato accessions (var. *cerasiforme*) were mainly classified into Group 4, except for LA1338, which was classified into Group 1. Group E consisted of 30 wild accessions belonging to *S. habrochaites*, *S. pennellii*, *S. cornerliomulleri*, *S. chilense*, *S. peruvianum*, *S. arcanum*, *S. huaylasense*, and *S. chmielewskii*. Group F consisted of 18 accessions including most accessions (13) of *S*. *pimpinellifolium* (except LA2633, which was classified into Group 1), 3 of *S*. *lycopersicum*, 1 of *S*. *galapagense*, and 1 of *S*. *cheesmaniae* (Figure 4a).

The genetic relationships of the accessions obtained using Genome Association and Prediction Integrated Tool (GAPIT) software were further confirmed based on a kinship plot and dendrogram (Figure 4b). The 290 accessions were divided into three clusters; Clusters 1 and 3 mainly consisted of *S*. *lycopersicum* and cherry tomato (var. *cerasiforme*) accessions, while most wild species and *S. pimpinellifolium* accessions that belonged to Groups 5 and 6, respectively, in the STRUCTURE analysis, were grouped into Cluster 2. In particular, the kinship heatmap showed a relatively high genetic similarity among wild species accessions in Cluster 2. Also, Clusters 1 and 3 were further divided into 3 (1-1, 1-2, and 1-3) and 2 (3-1 and 3-2) Subgroups, respectively. Subgroups 1–3 consisted of 55 accessions out of 63 accessions in pop 4, and 1 and 3 accessions belonging to pop 1 and 3, respectively, while there were mixtures of the remaining accessions in pop 1 and accessions in pop 2, 3, and 4 in Subgroups 1-1 and 1-2, respectively. Subgroup 3-1 consisted of 12 accessions of pop 1 and 2 accessions of pop 6, while in Subgroup 3-2, the three accessions of *S. pimpinellifolium*, *S. cheesmanii*, and *S. galapagense*, as well as five cherry tomatoes (var. *cerasiforme*) accessions were included.

### 2.9. GWAS for PMR

**Figure 5:**
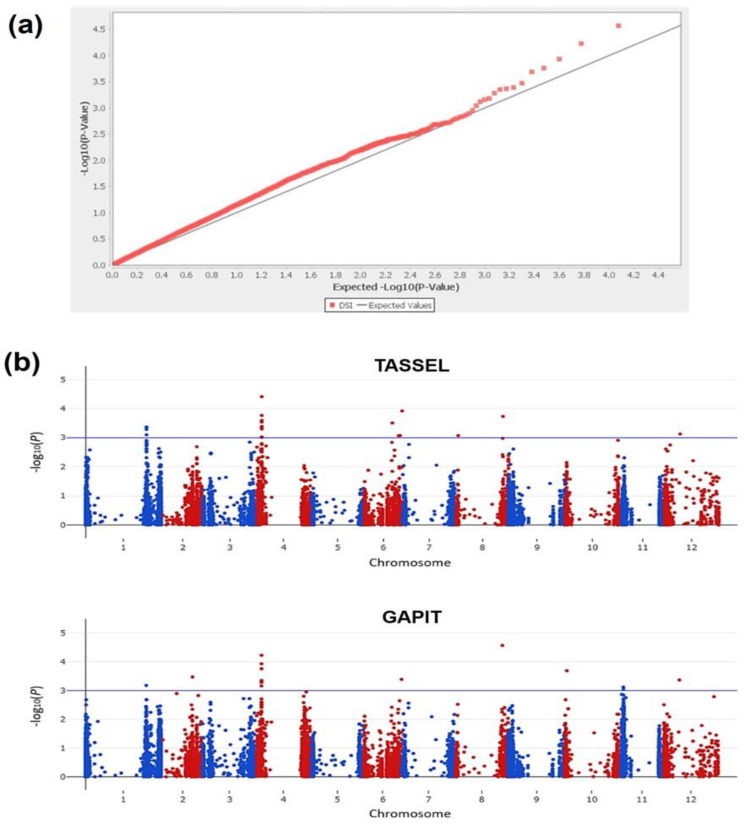
(**a**) Quantile-Quantile plots generated using mixed linear model (PC matrix + Kinship matrix) in TASSEL 5.0 software; (**b**) Manhattan plots generated using a mixed linear model (PC matrix + Kinship matrix) in TASSEL (**top**) and GAPIT (**bottom**) software for a genome-wide association study carried out for 11,912 single nucleotide polymorphism (SNP) loci obtained from 244 *S. lycopersicum* and 46 wild species accessions. The blue horizontal line represents −log_10_(*P*) = 3, and the SNPs above the blue line in the plot were selected as significant and highly correlated with powdery mildew resistance in tomato.

GWAS was first performed using 11,912 SNPs and 290 accessions, based on the mixed linear model (PC matrix + kinship matrix), in Traits Analysis by aSSociation, Evolution and Linkage (TASSEL). In the quantile-quantile plot, the actual tested *P*-value of the SNPs corresponding to −log_10_(*P*) was smaller than the predicted *p*-value, indicating a trait-genotype association (Figure 5a). Additional GWAS was performed using GAPIT, with the same analysis model as that used in TASSEL. The GWAS results obtained using the software have been presented as a Manhattan plot (Figure 5b).

**Figure 6:**
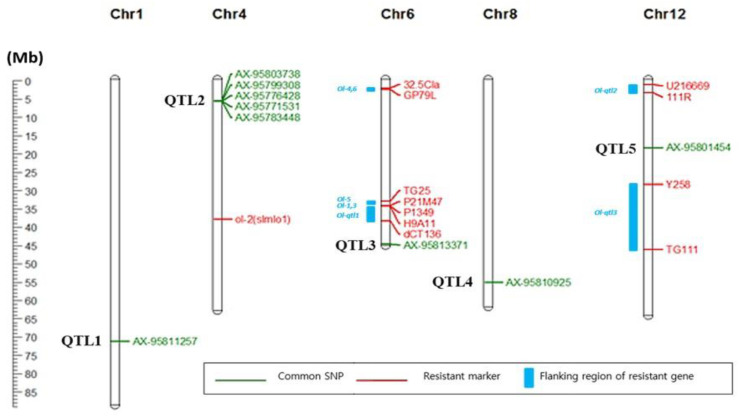
A schematic diagram of tomato chromosomes, with the genomic locations of the nine single nucleotide polymorphisms (SNPs, in green) significantly associated with powdery mildew resistance (PMR) in tomato depicted. The PMR loci and DNA markers identified in previous studies are shown in blue and red, respectively.

A total of 20 SNPs significantly associated with PMR at −log_10_(*P*) > 3 were detected on chromosomes 1, 4, 6, 8, and 12 in TASSEL, while 14 significant SNPs were found on chromosomes 1, 2, 4, 6, 8, 10, 11, and 12 in GAPIT (Table S4). Of these, 9 SNPs were commonly detected in both the analysis programs, which were located on chromosomes 1, 4, 6, and 8, and 12; these locations were designated as QTL-1, -2, -3, -4, and -5, respectively, in this study (Figure 6 and Table S5). Unlike other QTLs in which one SNP of common significance was detected, five common SNPs (AX-95803738, AX-95799308, AX-95776428, AX-95771531, and AX-95783448) were located in the QTL-2 region. Among the significant common SNPs, the most significant one predicted by GAPIT, AX-95771531 [−log_10_(*P*) = 4.41], was located in QTL-2 of chromosome 4, while the most significant one predicted by TASSEL, AX-95810925 [−log_10_(*P*) = 4.57], was located in QTL-4 of chromosome 8 (Table S5).

### 2.10. Putative Candidate Genes for PMR

**Figure 7:**
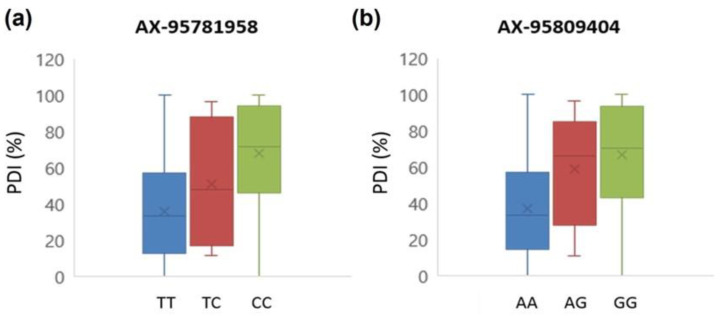
Box plots showing phenotypic distribution of the different genotypes of the SNPs (**a,b**). Blue and green represent the phenotypic distribution for the homozygous genotypes, while red represents that for the heterozygous genotype.

The regions, QTL-2 (chromosome 4, 5.87–6.07 Mb) and QTL-4 (chromosome 8, 57.17–57.27 Mb), which harbored AX-95771531 and AX-95810925, the most significant SNPs identified by GAPIT and TASSEL, respectively, were searched for the existence of candidate PMR genes. Assessing the annotation information from the SL4.0 (ITAG4.0) assembly of Sol Genomics Network (SGN, https://solgenomics.net/ accessed on 15 August 2022) revealed 22 genes in the QTL-2 region (Table S6). Among those, two nucleotide-binding site-leucine rich repeat (NBS-LRR) genes (Solyc04g015630.2.1 and Solyc04g015640.1.1), which are known as genes involved in disease resistance (R gene), were identified at the distances of 48,380 bp and 47,495 bp from the SNPs AX-95771531 and AX-95810925, respectively. No SNPs of the Axiom^®^ Tomato Genotyping Array were located within these NBS-LRR class genes. There were nine genes in the QTL-4 region, and the gene harboring AX-95810925 was another R gene, receptor-like serine/threonine-protein kinase (STPK) gene (Solyc08g074980.4.1) (Table S6). Two SNPs, AX-95809404 (A/G) and AX-95781958 (T/C) were located in the STPK gene exon. These SNPs were converted to CAPS markers and the tomato accessions used in the GWAS were genotyped for it, to assess the association between the marker genotype and phenotype (Table S7). In the case of AX-95781958, the accession carrying the marker genotypes of TT, TC, and CC showed PDI averages of 35.68%, 50.66%, and 67.90%, respectively, while the same were 36.71%, 58.99%, and 66.74% for the AX-95809404 marker genotypes of AA, AG, GG, respectively, thereby indicating significant differences in the level of PMR depending on the marker genotypes of the STPK gene (Figure 7).

## 3. Discussion

PM is a common disease that affects a wide range of host plants worldwide. To date, GWASes have been conducted on several crops such as barley, wheat, and oats, to explore PMR-related QTLs [26,27,28]. In tomato, PM is caused by *O*. *neolycopersici*.

Tomato disease resistance has been mostly found in wild species and has been utilized for breeding [29]. Genes exhibiting resistance to major diseases induced by various biotic stress factors such as tomato yellow leaf curl virus resistance genes *Ty1*,*3*, *Ty2*, *Ty4*, *ty5*, and *Ty6*; fusarium wilt resistance genes *I-1*, *I-2*, *I-3*, and *I-7*; late blight resistance genes *Ph-1*, *ph-2*, *ph-3*, and *Ph-5*; bacterial spot resistance genes *Rx-4* and *RXopJ4*; and nematode root-knot resistance gene *Mi-1* have been derived from wild species [30,31,32,33,34,35]. Except for the *Ol-6* gene, which is found in the breeding line but whose origin of tomato PMR is unknown, all other PMR genes are derived from wild species (*S. lycopersicum* var. *cerasiforme*, *S. peruvianum*, *S. habrochaites*, and *S. neorickii*). Our bioassay results also indicated that PMR is mainly found in wild species and appears very rarely in cultivated ones [5].

This is the first study to report PMR among the wild species accessions of *S. chilense* LA1963 and LA2932, *S. chmielewskii* LA2663, *S. cornerliomulleri* LA1274, *S. galapagense* LA1141, *S. pennellii* LA0716, LA1340, LA1272, LA1674, and LA1809, and *S. pimpinellifolium* LA2181 and LA2184, and thereby, highlights them as of important value for use as genetic resources for PMR breeding. According to Lindhout et al. [36], the wild species *S. chilense* and *S. peruvianum*, which belong to the *peruvianum* complex have a crossing barrier with cultivated species, whereas *S. pimpinellifolium*, *S. pennellii*, and *S. habrochaites*, which belong to the *esculentum* complex, have high cross-compatibility. Therefore, *S. pimpinellifolium* accessions LA2181 and LA2184, *S. pennellii* accessions LA0716, LA1272, LA1340, LA1674, and LA1809, and S*. habrochaites* accessions PI126445, PI134418, and PI308182, which have been identified as PM-resistant accessions in this study, can be used as a good material for crossbreeding with elite cultivars lines.

Similar to the previously reported tomato PMR gene (*Ol*)-associated marker test for the collected genetic resources, the resistance marker type was mostly found in the wild accessions that showed resistance, but not in the cultivars that showed sensitivity. This suggests that the previously reported PMR-linked markers can be utilized for MAS when introducing resistance genes from these wild species accessions into cultivars through crossbreeding. In addition, there was no resistance marker type found in the A1161, A1162, A1164, and A1216 lines, which are rare resistant cultivars. It can be speculated that the resistance of these cultivars is regulated by a new PMR gene(s) or allele that occurred during the domestication of tomato cultivars and has not been reported so far.

It can be observed from the results of this study that in some wild species lines, a band size different from the size expected for an existing PMR marker was not observed, or PCR amplification was not performed at all. This trend was observed regardless of species match with the accession used for resistance gene mapping. Cases of observation of amplification products at sizes different from the expected size have been reported in the study by Singh et al. [37], which screened diverse genetic resources for rice blast resistance gene(s) using existing resistance markers. It is presumed there was a nucleotide sequence variation in the primer sites or restriction enzyme recognition sites between the line used in this study and the line used for the existing resistance gene mapping. In addition, this non-specific amplification was mainly observed as most of the PMR-linked, non-gene-based markers tested in this study were CAPS converted from restriction fragment length polymorphism markers [6,7,8]. However, the test results of the two PMR gene-based ol-2 markers (M/Slmlo1, dCAPS_Slmlo1.1) produced only the amplification product of the expected size from all accessions, which demonstrates that gene-based marker development is important for more accurate and consistent MAS targeting various genetic resources.

The SNP detection probe for the Axiom^®^ Tomato Genotyping Array was developed based on the genome sequencing of a commercial F1 cultivar (*S. lycopersicum*) [21]. To the best of our knowledge, this is the first study that has reported the use of the Axiom^®^ Tomato Genotyping for SNP genotyping of diverse wild species tomatoes. In this study, the marker genotype mismatch between the CAPS/dCAPS assay and the SNP chip was mainly observed in wild species, which could be due to a genotyping error caused by a weak hybridization reaction of the probe to the wild species genome, and thereby, lowering of the SNP calling signal intensity. This assumption is supported by the result that wild species accessions tended to have low DQC, low call rate, and high heterozygosity rate, and also the fact that the lower the homology between the reference genome assembly, the wild species, and the probe sequence, the higher probability that the SNP-chip genotype was called out as missing or heterozygosity. Nevertheless, the clear distinction between species in the population structure analysis shows that Axiom^®^ Tomato Genotyping Array can be fully utilized for genome-wide SNP genotyping of various wild tomato species and further genetic analysis based on that. However, for reliable analysis results, rigid SNP filtering, such as the removal of SNPs with a high heterozygosity rate, as performed in this study, is essential.

In this study, 5 QTL regions associated with tomato PMR and 9 novel SNP markers located therein were finally selected by means of GWAS. Among these 9 SNPs, the maximum −log_10_(*P*) value was between 4 and 5, which is significantly low in comparison to that observed in other disease resistance GWAS cases, including PMR in barley [−log_10_(*P*) = 9.69] [27], blight resistance in pepper (11.23) [38], and gray leaf spot resistance in tomatoes (19.96) [39]. In general, a statistically high correlation analysis (significant *p*-value threshold = 5 × 10^–8^) using GWAS is possible when the gene of the target trait exists at a high frequency in the form of a common variant (allele) within a large population of core collection [40]. Two major factors lower the GWAS analysis ability of this study: the tomato PMR is a result of a rare allele with a low frequency in some wild species, and the resistance locus is also found at several genomic locations as QTLs [5]. Several recent studies have reported the strategies that could be adopted to set a genome-wide significant *p*-value threshold in GWAS, depending on the genetic structure and characteristics of the population (linkage disequilibrium, homogeneity, and ancestry) and the frequency of the target gene within the population [40,41].

In this study, genes annotated as NBS-LRR and receptor-like STPK were selected as potential candidate genes for PMR. The gene encoding the NBS-LRR protein is a representative disease resistance gene (R gene) that activates a pathogen-specific defense system together with receptor-like kinase [42,43,44]. In the QTL-2 region of chromosome 4 detected in this study, two NBS-LRR genes (PMR-NBS1 and PMR-NBS2) were clustered at very close positions, thereby indicating that this could be a potential genomic region involved in PMR. The previously reported PMR loci *Ol-4* and *Ol-6*, which induced rapid resistance to *O*. *neolycopersici* through a single cell hypersensitive reaction, have been also presumed to be a homolog to the *Mi-1* locus in which a number of R genes encoding NBS-LRR proteins form a cluster [5,6]. Other cases of the NBS-LRR gene clusters related to PMR include *Mla* in barley [45], *REN1* in grape (*Vitis vinifera*) [46], and *MtREP1* in alfalfa (*Medicago sativa*) [47].

In addition to NBS-LRR, receptor protein kinase, another protein taxon involved in plant disease resistance, and receptor-like STPK belonging to the receptor-like kinase family, are also involved in signal transduction processes required for disease resistance, through phosphorylation of serine and threonine residues [43]. In the case of the bacterial speck resistance gene *Pto* encoding serine/threonine kinase in tomatoes, Pto kinase recognizes the elicitor generated by the avrPto of the pathogen *Pseudomonas syringae* pv. tomato and activates another serine/threonine kinase, *Pti1*, in addition to interacting with the NBS-LRR gene, *Prf*, to induce resistance responses such as hypersensitivity reactions and oxidative bursts [48,49,50,51]. Furthermore, it has been shown that the receptor-like serine/threonine-protein kinase gene (*Stpk-V*) cloned from wheat (*Dasypyrum vilosum*) is located within the PMR locus *Pm21* and reduces haustoria formation of the PM pathogen *Blumeria graminis* f. sp. *tritici* [52]. In our study, the AX-95810925 in chromosome 8, which showed the highest −log_10_(*P*) value in TASSEL, was located in the intron region of the gene encoding RSTK. Moreover, there was a clear distinction in the phenotypic distribution based on the genotypes of SNPs AX-95781958 and AX-95809404 found in the exons of this gene, thereby suggesting that the SL-STPK gene may be a putative candidate for PMR in tomato.

PMR gene cloning is essential for rapid breeding processes based on the plant transformation. The first cloned PMR susceptibility (S) gene, *Slmlo1* for *ol-2*, is used for targeted mutagenesis such as RNAi-silencing and CRISPR/Cas9 technology [53,54,55]. These *Slmlo1*-transgenic plants demonstrated improved PMR and can be useful breeding resources for expedited development of new PMR cultivars. In addition, a putative gene (ShORR-1) for *Ol-1* was recently identified by complementary cDNA-amplified fragment length polymorphism (cDNA-AFLP) analysis of the resistant cultivar *S*. *habrochiates* G1.1560, carrying the *Ol-1*, which can be another target gene for genome editing mutagenesis [56].

In conclusion, PM bioassay and PMR-linked marker tests performed on a natural population with a diverse genetic background provided information about genetic resources that could be useful in MAS for PMR. Our GWAS identified nine significant SNPs from five novel QTL regions, which led to the selection of 3 PMR candidate genes from the two QTL regions where the most significant SNPs were located. The disease resistance locus information and resistance-related SNPs identified in this study will contribute to understanding the molecular basis of PMR and developing new PM-resistant cultivars. Further studies are needed to carry out gene-function analysis to verify the association of the putative candidate genes identified in this study with PMR.

## 4. Materials and Methods

### 4.1. Plant Materials

The genetic resources used for the PMR bioassay included a total of 295 accessions (Table S1). This accession panel was comprised of 51 accessions from 11 wild species and 244 accessions of cultivated tomato species (*S. lycopersicum*) obtained from the Asian Vegetable Research and Development Center (AVRDC), Tomato Germplasm Research Center at the University of California Davis (TGRC), Germplasm Resources Information Network, (GRIN), Gangneung-Wonju National University (GWNU), and Plant Genetics and Breeding Research Center at Pusan National University (PNU). Among them, four lines were reported as PM-resistant, including LA1969, LA0458 (Lv, *S. chilense*), LA2172 (carrying the PMR gene *Ol-4*, *S*. *peruvianum*), and PI247087 (*Ol-5*, *S*. *habrochaites*) [6,7,57,58], as well as Moneymaker (*S*. *lycopersicum*) and another accession reported as being susceptible to PM, PNU-PMS (*S*. *lycopersicum*), were used as controls for the bioassay [59].

### 4.2. Pathogen Identification

#### 4.2.1. Pathogen Isolation and Genomic DNA Extraction

A spore suspension was prepared for the isolation and identification of PM pathogens that occurred naturally in the greenhouse at Pusan National University in 2020. The PM spores were separated from those leaves of “Moneymaker” that showed symptoms of PM, by immersing them in double distilled water and then collecting the water after filtering it out using gauze. Thereafter, the suspension was placed in a 2.0 mL tube, centrifuged at 13,000× *g* for 1 min, following which the supernatant was discarded and the spores collected at the bottom were collected.

Thereafter, 400 μL of sodium dodecyl sulfate (SDS) was added to the spore pellet and incubated with it for 50 min, in a water bath maintained at 65 °C, to release the genomic DNA. To separate the cell debris and SDS solution from the DNA, 200 μL of 7.5 M ammonium acetate was added to the solution, mixed evenly, left on ice for 15 min, and centrifuged at 13,000× *g* for 10 min. Following that, 400 μL of the supernatant was collected and transferred to a new 1.5 mL tube. For DNA precipitation, 2.5 μL of glycogen (10 mg/mL) and 600 μL of isopropanol were added to the supernatant, and it was centrifuged at 13,000× *g* for 10 min. The obtained supernatant was discarded, and 300 μL of 70% ethanol was added to the generated DNA pellet, to remove the residual salt. The obtained solution was again centrifuged at 13,000× *g* for 10 min. After the DNA pellet was completely dried, it was resuspended in 60 μL of Tris-EDTA buffer, and quantified with a NanoDrop™ 1000 Spectrophotometer (Thermo Fisher Scientific, Waltham, MA, USA).

#### 4.2.2. Sequencing of the ITS Region of the Pathogen

Sequencing of the ITS region was performed to identify the isolated pathogen (named PNU-PM in this study). PM pathogen-specific primers PMITS1 (5′-TCGGACTGGCCTCAGGGAGA-3′) and PMITS2 (5′-TCACTCGCCGTTACTGAGGT-3′) [24] were used to amplify the corresponding ITS region sequence using PCR. The PCR mixture consisted of 5 μL of pathogen genomic DNA sample (20 ng/μL), 37.5 μL of double distilled water, 1 μL of dNTPs (10 mM, SolGent, Daejeon, Korea), 5 μL of 10× buffer (SolGent), 2.5 μL each of the 10 pmol forward and reverse primers, and 0.5 μL of Taq polymerase (5 U/mL, SolGent), mixed to prepare a total volume of 50 μL.

PCR was carried out using the Veriti™ 96-Well Thermal Cycler (Thermo Fisher Scientific) and the following PCR conditions; 1 cycle of pre-denaturation at 94 °C for 1 min, 35 cycles of denaturation at 94 °C for 1 min, annealing at 65 °C for 1 min, and extension at 72 °C for 2 min, followed by 1 cycle of final extension at 72 °C for 7 min. The PCR amplicons were electrophoresed on a 1% agarose gel, at 200 V for 45 min, and purified from the gel using the Expin™ Gel SV Gel Extraction Kit (GeneAll, Seoul, Korea). Direct sequencing of the purified PCR amplicons was performed using the dye-terminator method at Genotech (Daejeon, Korea).

#### 4.2.3. Pathogen Identification

The ITS region sequence of PNU-PM was analyzed for similarity to 27 ITS sequences of different fungal species used for phylogenic analysis in Kiss et al. [24] and the *L*. *taurica* ITS sequence (accession number: MH698492.1) obtained from NCBI (https://www.ncbi.nlm.nih.gov/ accessed on 15 August 2022), by means of pairwise/multiple alignments using Clustal W [60] in Molecular Evolutionary Genetics Analysis version X (MEGA version X) [61]. Phylogenetic analysis of the PM pathogens was based on the neighbor-joining method [62], with the Jukes–Cantor model [63] set as the nucleotide substitution model. The phylogenetic tree branch was created through 1000 iterations of the bootstrap method. In addition, the ITS sequence of PNU-PM was subjected to a BLASTn search (https://blast.ncbi.nlm.nih.gov/Blast.cgi accessed on 15 August 2022), to confirm the pathogen species with the highest concordance rate.

### 4.3. PM Bioassay

PM bioassay was performed in March 2020 in the greenhouse of Pusan National University. For a total of 348 accessions, 10 seeds per accession were sown into 50-cell trays filled with seedling medium (horticultural medium no. 2, Nongwoo Bio, Suwon, Korea) and the seedlings were grown under the conditions of 26 ± 5 °C and 40–70% relative humidity. The spore suspension for pathogen inoculation was prepared as described in Section 4.2.1. From 4 weeks after sowing, a spore suspension of 2.4 × 10^4^ spores/mL concentration was sprayed 3 times, at intervals of 2 weeks, using a manual sprayer, to the extent that the plants were soaked.

**Figure 8:**
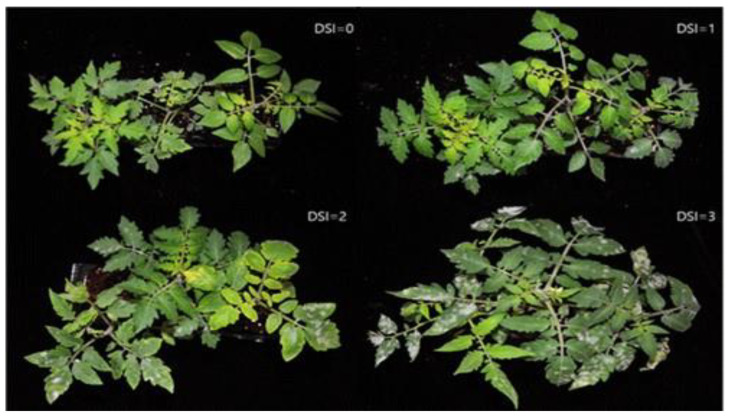
Disease severity rating scales used for assessing host plant resistance to powdery mildew in tomato. Disease severity index (DSI) was implemented based on visual rating scales of diseased leaf area (%) from the 1st to 6th true leaf. DSI = 0, 0%; DSI = 1, <10%; DSI = 2, 10–30%; and DSI = 3, >30%.

The disease severity index (DSI) was estimated by observing PM symptoms from 1 to 6 true leaves, in the 3rd week after completion of the last inoculation. The DSI was established based on the following criteria. 0: leaf area symptoms in up to 6 true leaves = 0%, 1: <10% leaf area symptoms, 2: 10–30% leaf area symptoms, 3: >30% leaf area symptoms (Figure 8). Later, the PDI was calculated according to Ullah et al. [64], as follows;

$$\mathrm{PDI}=\frac{\mathrm{Sum}\;\mathrm{of}\;\mathrm{DSI}\;\mathrm{of}\;\mathrm{individuals}}{\mathrm{Maximum}\;\mathrm{DSI}\;\times \;\mathrm{No}.\;\mathrm{of}\;\mathrm{individuals}}\times 100(\%)$$

For each accession, while referring to the PDI values of MM and PMS as controls, PDI < 10% was scored as resistance (R), PDI in the range of 10–30% as moderate resistance (MR), and PDI > 30% as susceptibility (S).

### 4.4. Evaluation of PMR-Linked Molecular Markers

A total of 56 accessions, including 22 that were highly resistant (R), 28 highly sensitive (S), and the PMR and PMS controls were genotyped for a total of 10 previously reported PMR-linked markers (Table 4). For PCR amplification, a PCR mixture of 10 μL total volume, containing 1 μL of template DNA (20 ng/μL), 6.7 μL of double distilled water, 0.2 μL of 10 mM dNTPs, 1 μL of 10× buffer, 0.5 μL each of the 10 pmol forward/reverse primers, and 0.1 μL Taq polymerase (5 U/μL), was used per sample.

PCR was performed using a Veriti™ 96-Well Thermal Cycler and the following conditions: 1 cycle of pre-denaturation at 95 °C for 1 min, 35 cycles of denaturation at 95 °C for 30 s, annealing at Tm (°C) in Table 4 for 30 s, and extension at 72 °C for 1 min, followed by a final extension at 72 °C for 7 min. In case of touchdown PCR, after pre-denaturation at 95 °C for 2 min, there were 10 cycles of denaturation at 94 °C for 15 s, annealing at 60 °C (–0.5 °C/cycle) for 30 s, and extension at 72 °C for 1 min, followed by 30 cycles of denaturation at 94 °C for 15 s, annealing at 55 °C for 30 s, and extension at 72 °C for 1 min, and final extension at 72 °C for 3 min. For sequence-characterized amplified region markers, the amplicons were electrophoresed immediately after PCR, while for CAPS markers, the amplicons were treated with the restriction enzyme, according to the manufacturer’s manual, and then electrophoresed. Electrophoresis was performed on a 2% agarose gel, at 190 V for 2 h, and after gel staining with ethidium bromide, the PCR amplicons were visualized under UV light using a gel image analysis system (CoreBio i-MAX™, Davinch-K, Seoul, Korea). The marker genotypes of the accessions used in the marker assay were determined by comparing the resistance-specific and sensitivity-specific band size information reported for each marker with the band size observed in this experiment (Table 4).

### 4.5. SNP Genotyping Using the Axiom^®^ Tomato Genotyping Array

For SNP genotyping, for use in the GWAS analysis, genomic DNA was extracted for the tomato accessions used in the PM bioassay. Fresh leaves of 3rd–4th true leave stage were collected from three individual seedlings per accession and pooled. Genomic DNA extraction was conducted using the SDS extraction method, with a partial modification, following which the quantity and quality of the purified DNA were measured as described in Section 4.2.1. All DNA samples were diluted to the final concentration of 20 ng/μL, and genotyping of a total of 51,214 SNP loci using the 55K Axiom^®^ Tomato Genotyping Array (Thermo Fisher Scientific) was performed by DNA Link Inc. (Seoul, Korea).

### 4.6. SNP Genotyping Data QC and SNP Filtering

In order to check whether the wild species accessions used in this study are well applied to the Axiom^®^ Tomato Genotyping Array, 125 cultivated species and 57 wild species accessions were selected and the DQC, call rate, and heterozygosity rate for the 51,214 raw SNPs in them were analyzed. Correlation analysis between these three factors was carried out by calculating the Pearson correlation coefficient and significance probability (*p*-value) using SPSS Statistics 25.0.0 (IBM Inc., Chicago, IL, USA).

To confirm sequence homology, DNA sequences corresponding to the 70 bp probe sequence for each of the 51,214 SNP loci of the 55K Axiom^®^ Tomato Genotyping Array were obtained from the reference genome assembly of *S*. *lycopersicum* and five wild species in NCBI (Table S8). Sequence alignment was performed using the Bowtie2 program [65], under the following conditions: maximum mismatches in seed alignment(–N) = 1, length of seed substrings(–L) = 20, and the interval between seed substrings w/r/t read len(i) = S, 1, 0.50. Then, for each SNP locus in the Axiom^®^ Tomato Genotyping Array, the correlation of the ratio of missing data and heterozygosity with the degree of variation between probe and reference genome sequence was calculated in the 169 accessions (125 from *S. lycopersicum*, 8 from *S. chilense*, 8 from *S. habrochaites*, 10 from *S. pennellii*, 18 from *S. pimpinellifolium*, and 3 from *S. arcanum* 3) that were used in SNP genotyping data QC.

### 4.7. SNP Conversion to CAPS and dCAPS Markers

To verify the reliability of the SNP genotyping results, six of the SNPs genotyped on the Axiom^®^ Tomato Genotyping Array were selected and converted into CAPS or dCAPS markers (Table 5). The physical location of the selected SNPs was searched for in the SL4.0 assembly of SGN, to extract the flanking sequence. For CAPS and dCAPS marker conversion, restriction enzymes capable of distinguishing polymorphisms were confirmed using NEB cutter v2.0 (http://nc2.neb.com/NEBcutter2/index.php accessed on 15 August 2022) and dCAPS finder 2.0 (http://helix.wustl.edu/dcaps/ accessed on 15 August 2022), respectively. The primers were designed using Primer3 v0.4.0. (https://bioinfo.ut.ee/primer3-0.4.0/ accessed on 15 August 2022). PCR, restriction enzyme treatment and electrophoresis were performed in the same manner as that for the CAPS marker, as described in Section 4.4, except that PCR was performed with annealing at 58 °C, in a non-touchdown method.

### 4.8. GWAS

#### 4.8.1. Accessions and SNP Filtering

GWAS for PMR was performed using the PM bioassay and SNP genotype data from 290 accessions (244 accessions of *S. lycopersicum* and 46 accessions of wild species) that showed relatively low standard deviation in the bioassay (Table S1). SNP filtering from the 51,214 raw SNPs was performed by applying the conditions of minor allele frequency > 5%, missing proportion <5%, and heterozygosity proportion <10% (only for wild species).

#### 4.8.2. Population Structure Analysis

Population structure analysis was performed based on the admixture model of STRUCTURE v2.3.4 [66] using the 290 accessions and the filtered SNPs selected for GWAS. In the K (number of population) range of 1 to 1000 burn-in periods and 10,000 Markov Chain Monte–Carlo (MCMC) for each K were repeated 10 times. To determine the optimal size of the population, the value of ad hoc statistics (∆K) was calculated using the Evanno method [67] with STRUCTURE HARVESTER (http://taylor0.biology.ucla.edu/structureHarvester/ accessed on 15 August 2022). The genetic relationship among the accessions was analyzed using the VanRaden kinship algorithm [68] on the R-based package GAPIT, and confirmed through a Kinship heatmap plot and dendrogram.

#### 4.8.3. GWAS Analysis

GWAS was performed based on the mixed linear model (PC matrix + kinship matrix) and applied to the analysis using two programs, Traits Analysis by aSSociation, Evolution and Linkage (TASSEL) 5.0 [69] and R-based package Genome Association and Prediction Integrated Tool (GAPIT) ver. 3 [70]. The results from both programs were visualized and confirmed with a Manhattan plot. From the analysis result, SNPs with a *p*-value < 0.001 were considered significant SNPs associated with PMR, and only SNPs that were common to both TASSEL and GAPIT software were reselected and used for subsequent analysis.

#### 4.8.4. Candidate Gene Selection

For the identification of putative candidate genes for PMR, genes located within 50 kb on each flaking region of the SNPs with the highest −log_10_(*P*) value in TASSEL and GAPIT were selected from the SL4.0 assembly (ITAG4.0) in SGN. The genes related to disease resistance were then selected as candidate genes based on the gene function annotation information.

Alle experimental procedures described in the section ‘4. Materials and Methods’ were schematically represented in Figure S1.

## Figures and Tables

**Table 1:** Results of genotyping for powdery mildew resistance (PMR) markers in powdery mildew-resistant tomato accessions, and their PMR genes inferred based on the marker genotype.

Accession	Species	PDI (%)	Expected PMR Locus	P1349 (*Ol-1*,*3*) ^1^	H9A11 (*Ol-1*,*3*)	M/Slmlo1 [*ol-2* (*Slmlo1*)]	dCAPS_ Slmlo1.1 [*ol-2* (*Slmlo1.1*)]	GP79L (*Ol-4*,*6*)	32.5Cla (*Ol-4*,*6*)	TG25 (*Ol-5*)	P21M47 (*Ol-5*, *Ol-qtl1*)	dCT136 (*Ol-qtl1*)	Y258 (*Ol-qtl3*)	TG111 (*Ol-qtl3*)
KNU17	*S. lycopersicum*	0.00	*ol-2* *(Slmlo1.1)*	300/200	450	197	200	1200 /1000	773	300	226/90	150	137	397
LA1141	*S. galapagense*	0.00		300/200	425	197	170/30	750/600	320/120	300	196/90	150	110/26	397
LA1272	*S. pennellii*	0.00		500/300 /200	450	197	170/30	1000	700	320	316/196/ 120	180	110	N/A
LA1274	*S. cornerliomulleri*	0.00	*Ol-5/* *Ol-qtl1*,*3*	500/300 /200	425	197	170/30	1100/ 1000	773/460/ 230	350	196/120	180	110/26	1000
LA1674	*S. pennellii*	0.00	*Ol-qtl1*,*3*	500/300 /200	425	197	170/30	1000	700	320	196/120	180	110/26	1000
LA1809	*S. pennellii*	0.00	*Ol-qtl1*,*3*	500/300 /200	450/425	197	170/30	1000	700	320	196/120	180	110/26	1000
LA1963	*S. chilense*	0.00		500/300 /200	425	197	170/30	1200/ 1000/900	773/460/320	350/300	316/196 /120	180/150	multi band	1000
LA2181	*S.pimpinellifolium*	0.00		300/200	430	197	170/30	1000/750	773	300	226/90	150	137	397
LA2663	*S. chmielewskii*	0.00	*Ol-5 /* *Ol-qtl1*,*3*	300/200	N/A	197	170/30	N/A ^2^	320/120	350	196/120	180	110/26	1000
LA2744	*S.peruvianum*	0.00	*Ol-qtl3*	500/450/300/200	450/430/420	197	170/30	1200/ 1000	773/570/ 470/320/ 260/220	N/A	196/120	180/150	110/26	1000
LA2932	*S. chilense*	0.00		300/200	450	197	170/30	1200/ 1100	773/460/ 320	310	316/196/ 120	180	multi band	1000
PI126445	*S. habrochaites*	0.00	*Ol-4*,*6/* *Ol-5/* *Ol-qtl3*	500	430	197	170/30	1200/ 1000	320/120	350	196/120	N/A	110/26	1000
A1161	*S. lycopersicum*	0.00		300/200	450	197	170/30	1000/750	773	300	226/90	150	137	397
A1162	*S. lycopersicum*	0.00		300/200	450	197	170/30	1000/750	773	300	226/90	150	137	397
A1216	*S. lycopersicum*	0.00		300/200	450	197	170/30	1000/750	773	300	226/90	150	137	397
KNU16	*S. lycopersicum*	4.76	*ol-2* *(Slmlo1.1*)	300/200	450	197	200	1100/750	773	300	226/90	150	137	397
LA0716	*S. pennellii*	4.76	*Ol-1*,*3/* *Ol-qtl1*,*3*	500	450	197	170/30	1000	320/120	320	196/120	180	110/26	1000
LA1340	*S. pennellii*	5.56	*Ol-qtl3*	300/200	450	197	170/30	1000	700	320	316/196/ 120	180	110/26	1000
LA2184	*S. pimpinellifolium*	5.56		300/200	450	197	170/30	750	773	300	226/90	150	137	397
PI308182	*S. habrochaites*	6.67	*Ol-5/* *Ol-qtl3*	500	430	197	170/30	1000	773/700/ 320/120	350	196/120	N/A	110/26	1000
PI134418	*S. habrochaites*	8.33	*Ol-1*,*3/* *Ol-5*	500	400	197	170/30	N/A	773/700/ 320/120	350	196/120	N/A	multi band	N/A
A1164	*S. lycopersicum*	9.52		300/200	450	197	170/30	1000/850/750	773	300	226/90	150	137	1400/397
LA0458 (*Lv*)	*S. chilense*	0.00		300/200	450	197	170/30	1100/1000	460/320	280	196/120	150	multi band	1000
LA1969 (*Lv*)	*S. chilense*	0.00	*Ol-qtl1*	300/200	450	197	170/30	1000	320/120	350	196/120	180	multi band	1000
LA2172 (*Ol-4*)	*S. peruvianum*	20.82	*Ol-4*,*6/* *Ol-5/* *Ol-qtl3*	300/200	N/A	197	170/30	1200/ 1000	320/120	350	196/120	150	110/26	1000
PI247087 (*Ol-5*)	*S. habrochaites*	40.00	*Ol-5*	500	425	197	170/30	1000	320/120	350	196/120	N/A	multi band	1000
PNU-PMS	*S. lycopersicum*	66.67		300/200	450	197	170/30	1000/750	773	300	226/90	150	137	397
Money Maker	*S. lycopersicum*	100.00		300/200	450	197	170/30	1000/750	773	300	226/90	150	137	397

^1^ The PMR locus or loci associated with the marker is indicated in the parentheses. ^2^ N/A: Missing band. For the remaining PMR loci, two flanking markers for each locus were tested and the accessions were assumed to possess the PMR locus if both the markers showed a genotype specific to PMR. For a single dominant locus, *Ol-1*,*3* was observed in LA0716 (*S. pennellii*) and PI134418 (*S. habrochaites*), while *Ol-4*,*6* was observed in the *Ol-4*-derived accessions LA2174 (*S*. *peruvianum*) and PI126445 (*S*. *habrochaites*). *Ol-5* was found in seven accessions, including LA2174 (*S*. *peruvianum*), LA1274 (*S*. *cornerliomulleri*), LA2663 (*S*. *chmielewskii*), PI126445, PI134418, and PI308182 (*S*. *habrochaites*), and PI247087 (*S*. *habrochaites*), which has been reported as an *Ol-5*-derived accession.

**Table 2:** The Dish QC (DQC), call rate, and heterozygosity rate of single nucleotide polymorphisms obtained upon genotyping of 182 tomato accessions using the Axiom^®^ Tomato Genotyping Array.

Species (Number of Accessions)	DQC ^1^	Call Rate (%)	Heterozygosity Rate (%)
*S. lycopersicum* (34)	0.95	96.29	5.43
*S. lycopersicum* (*habrochaites* ILs ^2^) (10)	0.96	96.56	4.29
*S. lycopersicum* (*lycopersicoides* ILs) (31)	0.99	95.76	5.85
*S. lycopersicum* var. *cerasiforme* (50)	0.79	95.57	7.44
*S. cheesmaniae* (1)	0.15	95.31	7.74
*S. galapagense* (1)	0.14	95.51	7.53
*S. pimpinellifolium* (18)	0.10	94.21	14.88
*S. neorickii* (2)	0.04	91.85	25.22
*S. chmielewskii* (2)	0.04	91.02	26.87
*S. habrochaites* (8)	0.03	91.00	33.44
*S. pennellii* (10)	0.03	90.88	31.76
*S. peruvianum* (2)	0.03	90.33	29.85
*S. chilense* (6)	0.04	90.56	31.12
*S. arcanum* (3)	0.04	91.61	24.68
*S. corneliomulleri* (3)	0.04	90.68	27.42
*S. huaylasense* (1)	0.04	90.06	28.10

^1^ Dish QC (DQC) measures the amount of overlap between two homozygous peaks created by non-polymorphic probes. The DQC value of 1 indicates no overlap, which implies a reliable genotyping result, whereas that of 0 indicates a complete overlap. ^2^ IL: introgression line

**Table 3:** Comparisons between the results from the Axiom^®^ Tomato Genotyping Array and the PCR-based marker cleaved amplified polymorphic sequence and derived cleaved amplified polymorphic sequence genotyping for six single nucleotide polymorphisms (SNPs).

Sample	Species	PDI (%)	SNPs					
			AX-95781451	AX-95767557	AX-95784118	AX-95773152	AX-95809776	AX-95814666
KNU17	*S. lycopersicum*	0.00	GG	CC	AA	AA(GG) ^1^	TT	CC
LA1141	*S. galapagense*	0.00	GG	CG(GG)	AA	GG	TT	CC
LA1272	*S. pennellii*	0.00	AG(AA)	GG	AA(AG)	AG(GG)	TT	CT(TT)
LA1274	*S. cornerliomulleri*	0.00	AA	GG(CG)	AG	AG(GG)	NA ^2^ (TT)	NA(CC)
LA1674	*S. pennellii*	0.00	NA(AA)	GG	AG(GG)	AG(GG)	AT(TT)	CT(TT)
LA1809	*S. pennellii*	0.00	AG(AA)	GG	AA	AG(GG)	AT(TT)	CT(TT)
LA1963	*S. chilense*	0.00	AG(AA)	GG	GG	AG(GG)	TT	CT(TT)
LA2181	*S. pimpinellifolium*	0.00	GG	CG(GG)	AA	AG(GG)	TT	CC
LA2663	*S. chmielewskii*	0.00	NA (AA)	CG(GG)	NA(GG)	AG(GG)	AT(TT)	NA(CC)
LA2744	*S. peruvianum*	0.00	NA(AA)	NA(GG)	AG(GG)	AG(GG)	AT(TT)	CT
LA2932	*S. chilense*	0.00	AG(AA)	GG	AG(GG)	AG(GG)	TT	CT(TT)
PI126445	*S. habrochaites*	0.00	AA	GG	AG	AG(GG)	AT(TT)	CT(TT)
A1161	*S. lycopersicum*	0.00	GG	CC	GG	AG(GG)	TT	CT
A1162	*S. lycopersicum*	0.00	GG	CC	GG	AG(GG)	TT	TT(CT)
A1216	*S. lycopersicum*	0.00	GG	CC	GG	GG	AA	CC
KNU16	*S. lycopersicum*	4.76	GG	CC	AA	AA	TT	CC
LA0716	*S. pennellii*	4.76	AG(AA)	GG	NA(AA)	AG(GG)	NA(TT)	CT(TT)
LA1340	*S. pennellii*	5.56	AG(AA)	GG	AA(AG)	AG(GG)	AT(TT)	CT(TT)
LA2184	*S. pimpinellifolium*	5.56	GG(AG)	CC	NA(GG)	AG(GG)	NA(TT)	CC
PI308182	*S. habrochaites*	6.67	AA	GG	AG(GG)	AG(GG)	AT(TT)	CT(TT)
PI134418	*S. habrochaites*	8.33	AA	GG	NA(AG)	AG(GG)	AT(TT)	CT(TT)
A1164	*S. lycopersicum*	9.52	GG	CC	GG	AG(GG)	TT	TT

^1^ The PCR-based marker genotypes are presented in parentheses when they did not match the genotyping results from the SNP chip array. ^2^ NA: Missing SNP call signal.

**Table 4:** List of DNA markers for powdery mildew resistance in tomato reported in previous studies. The markers used for the genotyping tomato germplasm were highly resistant or susceptible to *Oidium neolycopersici*.

Marker	Primer Sequence (5’-3’)	Location (Chr, bp) ^1^	Tm (°C)	Marker Type ^2^ (Enzyme)	Target Locus	Reference
P1349	F:TGCTAAGAATCAGAAACCACACCT	Chr6 (Long arm)	56	CAPS	*Ol-1*,*3*	Bai et al. [6]
	R:ACAACAAGCTGATCCACCTAAAGA	35,136,894–35,137,379		(*Xcm* I)		
H9A11	F:TGCTCTAACAAAATCACCAAAATC	Chr6 (Long arm)	52	SCAR	*Ol-1*,*3*	Bai et al. [6]
	R:AAATGGTCAAACAAAGTCTATTGAG	35,347,388–35,347,820				
M/SlMlo1	F:ACCCTTAAGAAACTAGGGCAAA	Chr4 (Long arm)	55	SCAR	*ol-2*	Bai et al. [10]
	R:ACCATCATGAACCCATGTCT	38,702,897–38,703,092			(*Slmlo1*)	
dCAPS_ SlMlo1.1	F:TATATAGAGAAATTCTGTAGATGTGATC	Chr4 (Long arm)	50	dCAPS	*ol-2*	Kim et al. [55]
	R:TGGATAACCGCGTAATAAGT	38,700,768–38,700,970		(*Bcl* I)	(*Slmlo1.1*)	
GP79L	F:CACTCAATGGGGGAAGCAAC	Chr6 (Short arm)	56	CAPS	*Ol-4*,*6*	Bai et al. [6]
	R:AATGGTAAACGAGCGGGACT	2,746,498–2,747,964		(*Apo* I)		
32.5Cla	F:ACAGAAACAAAGTGCCAAG	Chr6 (Short arm)	56	CAPS	*Ol-4*,*6*	Bai et al. [6]
	R:CCACCACCAAACAGGAGTGTG	2,389,981–2,390,754		(*Hinf* I)		
TG25	F:TAATTTGGCACTGCCGT	Chr6 (Long arm)	52	SCAR	*Ol-5*	Bai et al. [6]
	R:TTGTYATRTTGTGYTTATCG	33,814,128–33,814,425				
P21M47	F:TAACAATCTCGACCATAGTTCC	Chr6 (Long arm)	T.D ^3^	CAPS	*Ol-5*,	Faino et al. [8]
	R:CCATACCCGAATTTCCTTCC	35,035,703–35,036,018		(*Hae* III)	*Ol-qtl1*	
dCT136	F:CGAAGTGTCGGATCCGAAGGCTTT	Chr6 (Long arm)	T.D	dCAPS	*Ol-qtl1*	Faino et al. [8]
	R:AACACAATCGGAAAAAA	39,171,973–39,172,139		(*Xmn* I)		
Y258	F:GTAATTCCAAAAAGTGAGGT	Chr12 (Long arm)	50	CAPS	*Ol-qtl3*	Bai et al. [7]
	R:TTGCGTCTAGAGTTATTTT	29,161,948–29,162,084		(*Mbo* I)		
TG111	F:TGCCAACCCGGACAAAGA	Chr12 (Long arm)	54	SCAR	*Ol-qtl3*	Bai et al. [7]
	R:TGGGGAAGTGATTAGACAGGACA	47,133,362–47,133,758				

^1^ Physical location (bp) of the genomic region flanked by the forward and reverse primer set. Each primer sequence was blasted to the tomato reference genome SL2.40 (https://solgenomics.net/ accessed on 15 August 2022), to find its physical location. ^2^ CAPS: cleaved amplified polymorphic sequence; SCAR: sequence-characterized amplified region; dCAPS: derived cleaved amplified polymorphic sequence. ^3^ T.D: Touchdown PCR.

**Table 5:** The cleaved amplified polymorphic sequence (CAPS) and derived cleaved amplified polymorphic sequence (dCAPS) marker information designed to validate the single nucleotide polymorphism (SNP) genotyping results of the Axiom^®^ Tomato Genotyping Array.

Marker Name	Target SNP	Location ^1^ (Chr, bp)	Marker Type (Enzyme)	Sequence (5’-3’)	Expected Size SNP: (bp)
SNP1_ CAPS	AX-95781451	Chr1: 815,871	CAPS	F:TTACATTTAACTGTGACAAGCAGAT	G:172/54
			(*Hpy99* I)	R:AGTAGTTCATTTTCATTGCGAACT	A:226
SNP2_ dCAPS	AX-95767557	Chr2:3,942,590	dCAPS	F: ATTATGCCAACAACAAATCAGACAC	C:25/155
			(*Aci* I)	R:ACAATAGAACAAGAAGCTGAAAGAA	G:187
SNP3_ CAPS	AX-95784118	Chr4:6,054,103	CAPS	F:CTTACCACATAAACATAGGGATCTG	G:155/72
			(*Hinf* I)	R:GGACCCAAATAATCATCAAATCTAT	A:227
SNP4_ dCAPS	AX-95773152	Chr6:34,738,782	dCAPS	F:TGATCAATTAATTCCCGGAGATAG	G:174
			(*Rsa* I)	R:AAAGGGCTCAATCTTTTATTTGTAT	A: 25/149
SNP5_ CAPS	AX-95809776	Chr8:56,273,372	CAPS	F:ATTTAGTTTTCATGTGTCGATGAAT	A:35/130
			(*Apo* I)	R:TTCCTTTTAGCAATGTGATAGTTTT	T:165
SNP6_ CAPS	AX-95814666	Chr11:49,774,551	CAPS	F:CACTTTCATTAGATTCTTGTGGTCT	C:188/47
			(*Nde* I)	R:CCACTAAGGTATCAATCAATTTTGT	T:235

^1^ Physical locations based on the tomato reference assembly SL4.0 from the Sol Genomics Network (SGN, https://solgenomics.net/ accessed on 15 August 2022).

## Data Availability

Not applicable.

## Supplementary Materials

The following supporting information can be downloaded at: https://www.mdpi.com/article/10.3390/ijms232113610/s1, Table S1: Phenotypic data of 295 tomato accessions obtained from powdery mildew (PM) (*Oidium neolycopersici*) disease assay; Table S2: Dish QC (DQC), call rate, heterozygosity rate information for each sample of the 182 accessions used for assessing the quality of results for the single nucleotide polymorphism (SNP) genotyping by the 55K Axiom^®^ Tomato Genotyping Array; Table S3: Genotypes of 11,912 single nucleotide polymorphisms (SNPs) for the 290 tomato accessions, revealed using the 55K Axiom^®^ Tomato Genotyping Array; Table S4: Single nucleotide polymorphisms (SNPs) selected for significant association [−log_10_(*P*) > 3] with powdery mildew resistance (PMR) in tomato, upon carrying out a genome-wide association study (GWAS); Table S5: Genotypes in 290 tomato accessions for nine single nucleotide polymorphisms (SNPs) identified to be significantly associated with powdery mildew resistance (PMR) in the genome-wide association study (GWAS); Table S6: List of annotated genes in the tomato powdery mildew resistance (PMR)-associated QTL-2 (chromosome 4, 5.87–6.07 Mb) and QTL-4 (chromosome 8, 57.17–57.27 Mb) regions containing two significant SNPs, AX-95771531 and AX-95810925, respectively; Table S7: The genotyping results reconfirmed by converting chip single nucleotide polymorphisms (SNPs) AX-95809404 and AX-95781958 into cleaved amplified polymorphic sequence (CAPS) markers to the 290 accessions used in the genome-wide association study (GWAS); Table S8: Tomato reference genome assembly information used for similarity analysis with the probe sequences of the 55K Axiom^®^ Tomato Genotyping Array; Figure S1: A schematic representation of experimental procedure.
