# Supplementary material for: Germplasm Screening Using DNA Markers and Genome-Wide Association Study for the Identification of Powdery Mildew Resistance Loci in Tomato
Source: Int J Mol Sci. 2022 Nov 6;23(21):13610. doi: 10.3390/ijms232113610 (PMC9657208; doi:10.3390/ijms232113610)
Supplement: Supplementary file 1 [file ijms-23-13610-s001.zip › Table S2.pdf]

Table S2. Dish QC (DQC), call rate, heterozygosity rate information for each sample of 182 accessions used for single nucleotide polymorphism (SNP) quality control.

Sample	Species	DQC	Call rate (%)	Heterozygosity rate (%)
AVTO98039	*S. lycopersicum*	0.97	97.25	6.47
BT0902	*S. lycopersicum*	0.77	96.80	6.81
BT0909	*S. lycopersicum*	0.96	95.63	8.57
BT0911	*S. lycopersicum*	0.78	92.25	9.77
BT0912	*S. lycopersicum*	1.00	95.88	9.23
Edkawi	*S. lycopersicum*	1.00	97.76	2.45
Fla.8233	*S. lycopersicum*	0.97	98.21	2.91
Floradel	*S. lycopersicum*	1.00	96.87	3.75
Florida MH-1	*S. lycopersicum*	1.00	97.96	2.52
Green Doctor	*S. lycopersicum*	0.79	97.06	6.55
King Humbert#1	*S. lycopersicum*	0.99	96.62	3.30
KNU2	*S. lycopersicum*	0.94	91.56	13.16
KNU11	*S. lycopersicum*	0.73	92.07	12.56
KNU14-1	*S. lycopersicum*	0.97	95.27	4.90
KNU15	*S. lycopersicum*	0.98	95.83	6.07
KNU16	*S. lycopersicum*	0.96	96.30	3.62
KNU17	*S. lycopersicum*	0.96	97.93	4.53
LA0172	*S. lycopersicum var. cerasiforme*	0.98	98.12	2.75
LA0292	*S. lycopersicum var. cerasiforme*	0.95	95.31	5.78
LA0373	*S. pimpinellifolium*	0.03	94.89	15.99
LA0407	*S. habrochaites*	0.03	90.94	34.14
LA0446	*S. peruvianum*	0.03	90.16	30.54
LA0716	*S. pennellii*	0.02	91.16	32.07
LA0722	*S. pimpinellifolium*	0.01	93.45	12.59
LA1028	*S. chmielewskii*	0.04	92.01	24.53
LA1141	*S. galapagense*	0.14	95.51	7.53
LA1208	*S. lycopersicum var. cerasiforme*	0.98	94.21	5.77
LA1218	*S. lycopersicum var. cerasiforme*	0.95	97.86	2.92
LA1228	*S. lycopersicum var. cerasiforme*	0.90	94.01	6.49
LA1237	*S. pimpinellifolium*	0.16	96.53	6.58
LA1246	*S. pimpinellifolium*	0.02	95.61	8.38
LA1269	*S. pimpinellifolium*	0.95	95.89	4.21
LA1272	*S. pennellii*	0.03	90.89	31.09
LA1274	*S. corneliomulleri*	0.04	90.88	26.52
LA1283	*S. corneliomulleri*	0.04	90.36	29.05
LA1290	*S. lycopersicum var. cerasiforme*	0.93	93.26	7.13

LA1307	*S. lycopersicum var. cerasiforme*	0.83	96.88	4.96
LA1314	*S. lycopersicum var. cerasiforme*	0.73	95.69	5.70
LA1322	*S. neorickii*	0.05	91.81	25.76
LA1338	*S. lycopersicum var. cerasiforme*	0.40	90.43	23.50
LA1340	*S. pennellii*	0.02	91.59	30.38
LA1346	*S. arcanum*	0.04	91.32	25.20
LA1406	*S. cheesmaniae*	0.15	95.31	7.74
LA1455	*S. lycopersicum var. cerasiforme*	0.94	90.91	14.07
LA1456	*S. lycopersicum var. cerasiforme*	0.19	96.61	5.82
LA1457	*S. lycopersicum var. cerasiforme*	0.32	95.23	6.73
LA1461	*S. lycopersicum var. cerasiforme*	0.94	98.06	2.82
LA1464	*S. lycopersicum var. cerasiforme*	0.95	96.25	3.84
LA1482	*S. lycopersicum var. cerasiforme*	0.97	97.37	3.19
LA1512	*S. lycopersicum var. cerasiforme*	0.94	98.18	2.86
LA1542	*S. lycopersicum var. cerasiforme*	0.94	94.20	9.21
LA1545	*S. lycopersicum var. cerasiforme*	0.95	97.19	3.26
LA1547	*S. pimpinellifolium*	0.02	89.87	27.80
LA1549	*S. lycopersicum var. cerasiforme*	0.89	95.78	4.30
LA1569	*S. lycopersicum var. cerasiforme*	0.89	98.07	2.96
LA1578	*S. pimpinellifolium*	0.00	94.00	14.45
LA1582	*S. pimpinellifolium*	0.01	94.23	13.91
LA1589	*S. pimpinellifolium*	0.00	95.76	9.47
LA1617	*S. pimpinellifolium*	0.04	93.23	12.84
LA1620	*S. lycopersicum var. cerasiforme*	0.85	96.89	7.37
LA1621	*S. lycopersicum var. cerasiforme*	0.88	91.30	12.08
LA1623	*S. lycopersicum var. cerasiforme*	0.95	97.82	2.84
LA1632	*S. lycopersicum var. cerasiforme*	0.95	97.87	2.84
LA1654	*S. lycopersicum var. cerasiforme*	0.98	94.13	9.41
LA1656	*S. pennellii*	0.03	90.97	32.08
LA1668	*S. lycopersicum var. cerasiforme*	0.95	97.87	2.89
LA1674	*S. pennellii*	0.02	91.38	30.72
LA1701	*S. lycopersicum var. cerasiforme*	0.73	91.17	19.41
LA1712	*S. lycopersicum var. cerasiforme*	0.89	91.99	10.29
LA1732	*S. pennellii*	0.02	89.68	33.71
LA1777	*S. habrochaites*	0.04	91.30	29.46
LA1809	*S. pennellii*	0.03	91.23	31.41
LA1912	*S. pennellii*	0.02	91.35	29.93
LA1930	*S.chilense*	0.03	90.58	29.57
LA1941	*S. pennellii*	0.02	91.23	31.31
LA1946	*S. pennellii*	0.02	89.37	34.94
LA1953	*S. lycopersicum var. cerasiforme*	0.84	97.79	3.35

LA1963	*S.chilense*	0.03	91.28	28.84
LA2076	*S. lycopersicum var. cerasiforme*	0.98	97.68	2.97
LA2077	*S. lycopersicum var. cerasiforme*	0.98	97.81	3.30
LA2078	*S. lycopersicum var. cerasiforme*	0.99	97.99	2.81
LA2093	*S. pimpinellifolium*	0.04	96.27	7.52
LA2099	*S. habrochaites*	0.05	90.88	33.91
LA2126A	*S. lycopersicum var. cerasiforme*	0.89	97.28	3.55
LA2131	*S. lycopersicum var. cerasiforme*	0.82	97.57	4.14
LA2135	*S. lycopersicum var. cerasiforme*	0.98	96.02	4.77
LA2137	*S. lycopersicum var. cerasiforme*	0.22	95.94	6.80
LA2163	*S. arcanum*	0.03	91.58	23.43
LA2181	*S. pimpinellifolium*	0.01	96.53	8.73
LA2184	*S. pimpinellifolium*	0.04	94.11	13.50
LA2185	*S. arcanum*	0.03	91.95	25.42
LA2190	*S. neorickii*	0.04	91.89	24.69
LA2308	*S. lycopersicum var. cerasiforme*	0.92	90.06	20.60
LA2312	*S. lycopersicum var. cerasiforme*	0.99	95.69	4.12
LA2411	*S. lycopersicum var. cerasiforme*	0.71	98.48	2.70
LA2446	*S. lycopersicum*	1.00	97.57	3.54
LA2533	*S. pimpinellifolium*	0.31	91.44	40.22
LA2561	*S. huaylasense*	0.04	90.06	28.10
LA2626	*S. lycopersicum var. cerasiforme*	0.80	97.31	6.16
LA2632	*S. lycopersicum var. cerasiforme*	0.37	91.13	28.24
LA2633	*S. lycopersicum var. cerasiforme*	0.16	96.65	5.56
LA2660	*S. lycopersicum var. cerasiforme*	0.01	89.86	19.56
LA2663	*S. chmielewskii*	0.04	90.04	29.21
LA2664	*S. lycopersicum var. cerasiforme*	0.99	98.55	2.40
LA2675	*S. lycopersicum var. cerasiforme*	0.21	94.06	7.33
LA2744	*S. peruvianum*	0.04	90.50	29.16
LA2779	*S.chilense*	0.03	91.38	28.43
LA2792	*S. lycopersicum var. cerasiforme*	0.43	90.78	30.45
LA2845	*S. lycopersicum var. cerasiforme*	0.85	98.14	3.07
LA2852	*S. pimpinellifolium*	0.09	94.65	17.37
LA2880	*S.chilense*	0.04	90.97	30.77
LA2930	*S.chilense*	0.03	89.98	33.50
LA2932	*S.chilense*	0.05	89.19	35.62
LA3043	*S. lycopersicum*	1.00	98.48	3.39
LA3044	*S. lycopersicum*	1.00	98.19	2.59
LA3045	*S. lycopersicum*	1.00	97.99	3.04
LA3047	*S. lycopersicum*	1.00	98.63	2.62
LA3051	*S. lycopersicum*	0.99	93.84	6.41

LA3136	*S. lycopersicum var. cerasiforme*	0.81	91.50	10.51
LA3137	*S. lycopersicum var. cerasiforme*	0.98	98.13	3.95
LA3246	*S. lycopersicum*	1.00	93.21	10.34
LA3267	*S. lycopersicum*	1.00	97.90	3.00
LA3344	*S. lycopersicum (lycopersicoides ILs)*	0.97	97.07	2.76
LA3345	*S. lycopersicum (lycopersicoides ILs)*	0.95	95.38	10.59
LA3736	*S. lycopersicum*	1.00	94.46	5.27
LA3866	*S. lycopersicum (lycopersicoides ILs)*	0.97	97.38	2.52
LA3867	*S. lycopersicum (lycopersicoides ILs)*	0.97	98.53	2.05
LA3869	*S. lycopersicum (lycopersicoides ILs)*	0.97	96.61	3.20
LA3870	*S. lycopersicum (lycopersicoides ILs)*	0.92	92.59	7.90
LA3871	*S. lycopersicum (lycopersicoides ILs)*	0.97	98.85	1.94
LA3874	*S. lycopersicum (lycopersicoides ILs)*	0.96	98.12	2.25
LA3875	*S. lycopersicum (lycopersicoides ILs)*	1.00	98.54	2.62
LA3878	*S. lycopersicum (lycopersicoides ILs)*	0.96	97.92	2.30
LA3879	*S. lycopersicum (lycopersicoides ILs)*	0.95	97.91	2.56
LA3882	*S. lycopersicum (lycopersicoides ILs)*	0.96	92.86	7.52
LA3883	*S. lycopersicum (lycopersicoides ILs)*	0.96	95.39	3.82
LA3886	*S. lycopersicum (lycopersicoides ILs)*	0.97	96.11	3.23
LA3889	*S. lycopersicum (lycopersicoides ILs)*	0.97	96.28	3.34
LA3892	*S. lycopersicum (lycopersicoides ILs)*	0.97	98.79	1.94
LA3893	*S. lycopersicum (lycopersicoides ILs)*	0.94	97.31	3.24
LA3918	*S. lycopersicum (habrochaites ILs)*	0.99	98.75	2.25
LA3919	*S. lycopersicum (habrochaites ILs)*	0.99	93.25	8.35
LA3920	*S. lycopersicum (habrochaites ILs)*	1.00	97.22	4.39
LA3925	*S. lycopersicum (habrochaites ILs)*	0.99	95.61	3.75
LA3928	*S. lycopersicum (habrochaites ILs)*	0.98	94.21	15.37
LA3951	*S. lycopersicum (habrochaites ILs)*	1.00	95.73	4.06
LA3960	*S. lycopersicum (habrochaites ILs)*	0.99	94.07	6.04
LA3975	*S. lycopersicum (habrochaites ILs)*	1.00	98.30	2.42
LA3997	*S. lycopersicum (habrochaites ILs)*	0.99	94.40	5.74
LA3999	*S. lycopersicum (habrochaites ILs)*	1.00	96.07	6.11
LA4024	*S. lycopersicum*	0.99	98.20	2.60
LA4230	*S. lycopersicum (lycopersicoides ILs)*	0.98	97.51	3.50
LA4239	*S. lycopersicum (lycopersicoides ILs)*	0.94	98.21	2.20
LA4240	*S. lycopersicum (lycopersicoides ILs)*	0.97	91.96	8.79
LA4244	*S. lycopersicum (lycopersicoides ILs)*	0.97	97.22	2.87
LA4245	*S. lycopersicum (lycopersicoides ILs)*	1.00	95.38	13.63
LA4246	*S. lycopersicum (lycopersicoides ILs)*	0.97	97.22	3.12
LA4247	*S. lycopersicum (lycopersicoides ILs)*	0.96	96.31	3.27
LA4257	*S. lycopersicum (lycopersicoides ILs)*	0.93	90.69	14.51

LA4264	*S. lycopersicum (lycopersicoides ILs)*	0.97	98.60	1.99
LA4266	*S. lycopersicum (lycopersicoides ILs)*	0.97	95.54	3.67
LA4270	*S. lycopersicum (lycopersicoides ILs)*	0.97	95.58	4.26
LA4271	*S. lycopersicum (lycopersicoides ILs)*	0.97	98.56	2.08
LA4279	*S. lycopersicum (lycopersicoides ILs)*	0.98	98.39	2.09
LA4282	*S. lycopersicum (lycopersicoides ILs)*	0.97	96.59	3.34
PI126434	*S. corneliomulleri*	0.03	90.80	26.67
PI126445	*S. habrochaites*	0.03	91.16	32.60
PI126925	*S. pimpinellifolium*	0.01	94.50	17.41
PI127827	*S. habrochaites*	0.03	90.23	34.94
PI128216	*S. pimpinellifolium*	0.00	96.22	9.20
PI134417	*S. habrochaites*	0.03	91.02	34.93
PI134418	*S. habrochaites*	0.03	91.30	33.52
PI180721	*S. lycopersicum*	1.00	98.46	2.42
PI205037	*S. lycopersicum*	0.99	96.62	3.14
PI254655	*S. lycopersicum*	0.69	91.26	12.45
PI296363	*S. lycopersicum*	1.00	93.03	7.70
PI308182	*S. habrochaites*	0.03	91.14	34.04
SAL1875	*S. pimpinellifolium*	0.01	88.64	27.63
2K6-8040	*S. lycopersicum*	0.95	98.13	2.83
2K6-8201	*S. lycopersicum*	0.97	98.62	2.84
Trujillo	*S. lycopersicum var. cerasiforme*	0.73	97.35	4.25
Walter	*S. lycopersicum*	1.00	98.18	3.31
