# Supplementary material for: Germplasm Screening Using DNA Markers and Genome-Wide Association Study for the Identification of Powdery Mildew Resistance Loci in Tomato
Source: Int J Mol Sci. 2022 Nov 6;23(21):13610. doi: 10.3390/ijms232113610 (PMC9657208; doi:10.3390/ijms232113610)
Supplement: Supplementary file 1 [file ijms-23-13610-s001.zip › Table S4.pdf]

Table S4. Single nucleotide polymorphisms (SNPs) selected for significant association ($-\log_{10}(P) > 3$) with the powdery mildew resistance (PMR) in tomato, upon carrying out a genome-wide association study.

SNP Marker[a]	SNP allele[b] (major/ minor)	Position[c]		$-\log_{10}(P)$		Gene annotation[d]		FDR adjusted *P*-values	R^2 (%)[e]
		Chr	bp	GAPIT	TASSEL	Gene ID	Gene function		
AX-95811257	T/C	1	72,655,237	3.36	3.18	Solyc01g009700.3.1	NL0E	0.4742	5.22%
AX-95805394	C/T	1	72,661,165	3.29	-	Intergenic region	-	0.4742	3.71%
AX-95777655	C/A	1	72,696,985	3.10	-	Solyc01g080870.3.1	Protein NRT1/ PTR FAMILY 7.3	0.5635	3.88%
AX-95779967	T/C	1	72,697,146	3.36	-	Solyc01g080870.3.1	Protein NRT1/ PTR FAMILY 7.3	0.4742	4.43%
AX-95804580	C/T	2	37,742,382	-	3.47	Intergenic region	-	0.9167	4.43%
AX-95803738	C/T	4	5,920,027	3.54	3.16	Intergenic region	-	0.4742	5.31%

AX-95788484	G/A	4	5,997,381	3.02	-	Solyc04g015690.4.1	Insulinase (Peptidase family M16) family protein	0.5732	4.32%
AX-95799308	A/G	4	6,001,287	3.39	3.35	Solyc04g015730.3.1	Hypothetical protein	0.4742	5.66%
AX-95776428	T/C	4	6,006,978	3.77	3.28	Intergenic region	-	0.4742	5.51%
AX-95799144	C/T	4	6,007,262	3.35	-	Solyc04g015700.1.1	Hypothetical protein	0.4742	4.43%
AX-95773950	G/A	4	6,008,475	3.03	-	Solyc04g015705.1.1	Unknown protein	0.5732	4.10%
AX-95771531	T/C	4	6,009,028	4.41	3.93	Solyc04g015710.3.1	Dentin sialophosphoprotein-like protein	0.4648	6.55%
AX-95806639	G/T	4	6,009,194	3.59	-	Solyc04g015710.3.1	Dentin sialophosphoprotein-like protein	0.4742	4.11%
AX-95790579	C/G	4	6,022,767	-	4.23	Intergenic region	-	0.9167	7.11%
AX-95783448	C/A	4	6,023,073	3.30	3.76	Solyc04g015730.3.1	Hypothetical protein	0.4742	6.53%
AX-95775687	G/C	6	34,025,307	3.51	-	Solyc06g059750.3.1	Transcriptional corepressor SEUSS	0.4742	4.09%

AX-95797861	A/C	6	41,603,822	3.07	-	Intergenic region	-	0.5635	4.09%
AX-95786699	T/C	6	43,282,486	3.07	-	Intergenic region	-	0.5635	4.41%
AX-95813371	G/A	6	45,685,033	3.92	3.39	Solyc06g084210.3.1	Sec24-like transport protein	0.4742	5.71%
AX-95790369	T/A	8	2,923,332	3.07	-	Intergenic region	-	0.5635	4.31%
AX-95810925	A/G	8	56,274,173	3.73	4.57	Solyc08g074980.4.1	Receptor-like serine/threonine-protein kinase	0.4742	7.68%
AX-95789984	A/G	10	2,629,440	-	3.69	Solyc10g150103.1.1	Unknown protein	1.0000	6.31%
AX-95780990	T/C	11	5,353,985	-	3.12	Solyc11g012540.2.1	F-box/kelch-repeat protein	1.0000	5.20%
AX-95813498	A/G	11	5,505,866	-	3.04	Solyc11g012740.2.1	Regulation of nuclear pre-mRNA domain-containing protein 1B-like	1.0000	4.99%
AX-95801454	G/A	12	18,875,528	3.13	3.37	Intergenic region	-	0.5635	5.65%

[a]SNP markers highlighted in red are significant SNPs commonly identified from TASSEL and GAPIT.

[b]Minor alleles are the allele favorable for the resistance.

[c]Positions were determined based on the 'Heinz 1706' reference genome assembly SL2.40 from the Sol Genomics Network (SGN, https://solgenomics.net/)..

[d]Gene annotation information were obtained from 'Heinz 1706' reference genome assembly SL4.0 (ITAG4.0) from the SGN.

[e]Proportion of phenotypic variance explained by SNP.
