# Supplementary material for: Germplasm Screening Using DNA Markers and Genome-Wide Association Study for the Identification of Powdery Mildew Resistance Loci in Tomato
Source: Int J Mol Sci. 2022 Nov 6;23(21):13610. doi: 10.3390/ijms232113610 (PMC9657208; doi:10.3390/ijms232113610)
Supplement: Supplementary file 1 [file Figure S1.pdf › Figure S1.pdf]

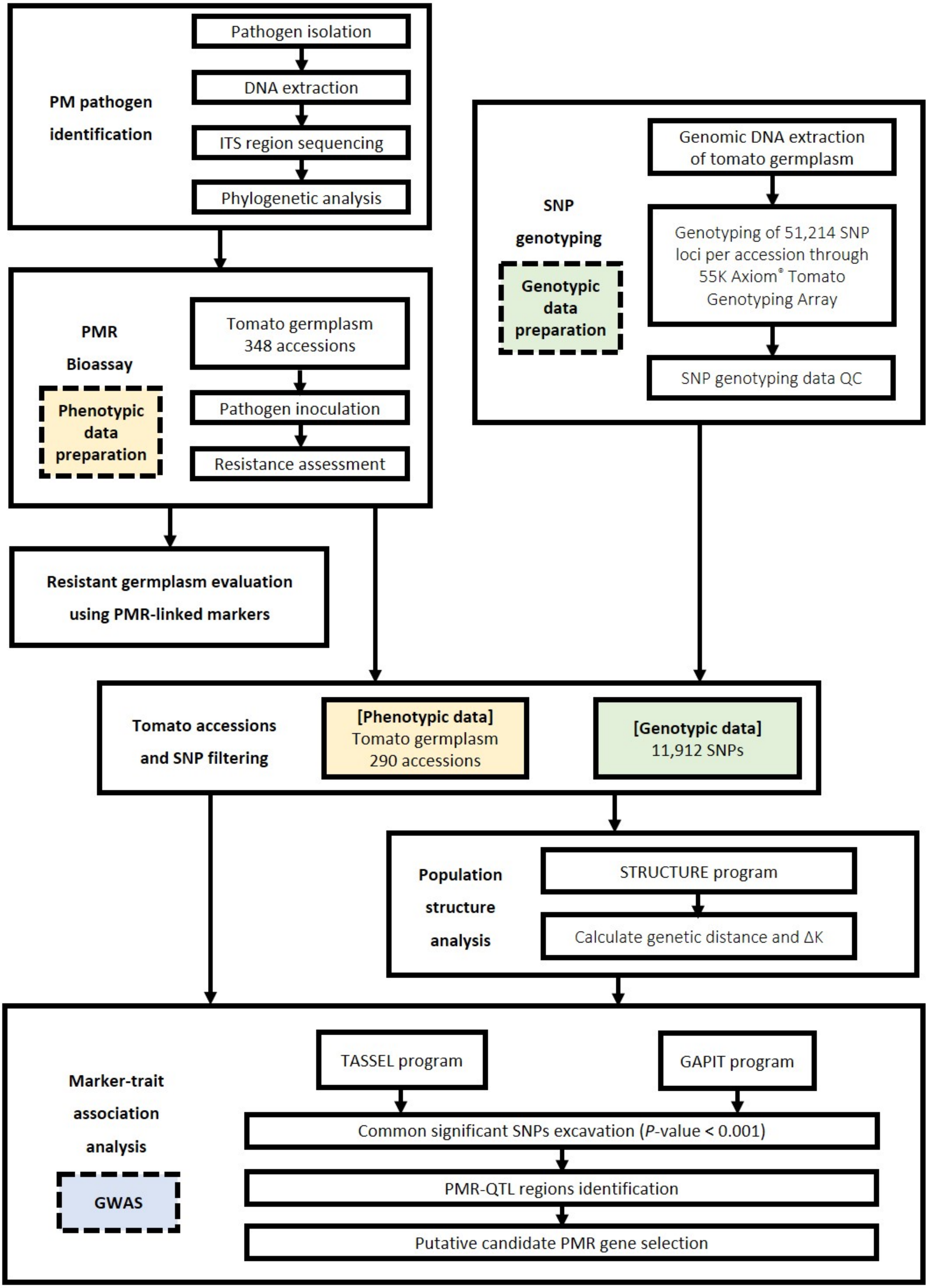
PM pathogen identification
Pathogen isolation
DNA extraction
ITS region sequencing
Phylogenetic analysis
PMR Bioassay
Phenotypic data preparation
Tomato germplasm 348 accessions
Pathogen inoculation
Resistance assessment
Resistant germplasm evaluation using PMR-linked markers
SNP genotyping
Genotypic data preparation
Genomic DNA extraction of tomato germplasm
Genotyping of 51,214 SNP loci per accession through 55K Axiom® Tomato Genotyping Array
SNP genotyping data QC
Tomato accessions and SNP filtering
[Phenotypic data] Tomato germplasm 290 accessions
[Genotypic data] 11,912 SNPs
Population structure analysis
STRUCTURE program
Calculate genetic distance and ΔK
Marker-trait association analysis
GWAS
TASSEL program
GAPIT program
Common significant SNPs excavation (P-value < 0.001)
PMR-QTL regions identification
Putative candidate PMR gene selection
