# Supplementary material for: Germplasm Screening Using DNA Markers and Genome-Wide Association Study for the Identification of Powdery Mildew Resistance Loci in Tomato
Source: Int J Mol Sci. 2022 Nov 6;23(21):13610. doi: 10.3390/ijms232113610 (PMC9657208; doi:10.3390/ijms232113610)
Supplement: Supplementary file 1 [file ijms-23-13610-s001.zip › Table S6.pdf]

Table S6. List of annotated genes in tomato powdery mildew resistance (PMR)-associated QTL-2 (Chr.4, 5.87-6.07 Mb) and QTL-4 region (Chr. 8, 57.17-57.27 Mb) containing two significant SNPs AX-95771531, AX-95810925, respectively.

QTL	Annotated gene	Description	Location[a]
QTL2	Solyc04g015580.3.1	Arm repeat superfamily protein	SL4.0ch04:5898847..5912928 (+ strand)
	Solyc04g150111.1.1	ozone-responsive stress-like protein	SL4.0ch04:5915991..5920041 (+ strand)
	Solyc04g015590.4.1	Bifunctional polymyxin resistance protein	SL4.0ch04:5920967..5924001 (+ strand)
	Solyc04g015600.3.1	Leucine-rich repeat receptor-like tyrosine-protein kinase	SL4.0ch04:5925036..5928968 (- strand)
	Solyc04g015610.3.1	DUF642 domain-containing protein	SL4.0ch04:5951876..5954495 (+ strand)
	Solyc04g015620.3.1	DUF642 domain-containing protein	SL4.0ch04:5956173..5958700 (+ strand)
	Solyc04g015630.2.1	NBS-LRR resistance-like protein	SL4.0ch04:5958826..5960648 (- strand)
	Solyc04g015640.1.1	Disease resistance protein rga3-like	SL4.0ch04:5960830..5961533 (- strand)
	Solyc04g015635.2.1	zinc finger BED domain-containing protein RICESLEEPER 2-like	SL4.0ch04:5964230..5966957 (- strand)
	Solyc04g015660.3.1	Serine/threonine-protein phosphatase 7 long form-like protein	SL4.0ch04:5975854..5979767 (+ strand)
	Solyc04g015680.3.1	insulin degrading enzyme	SL4.0ch04:5987660..6008485 (+ strand)
	Solyc04g015690.4.1	Insulinase (Peptidase family M16) family protein	SL4.0ch04:6009299..6036845 (+ strand)

	Solyc04g015700.1.1	hypothetical protein	SL4.0ch04:6038345..6038605 (- strand)
	Solyc04g015705.1.1	Unknown protein	SL4.0ch04:6038617..6039791 (- strand)
	Solyc04g015710.3.1	dentin sialophosphoprotein-like protein	SL4.0ch04:6040060..6044288 (- strand)
	Solyc04g015720.2.1	Unknown protein	SL4.0ch04:6045160..6048157 (- strand)
	Solyc04g015730.3.1	hypothetical protein	SL4.0ch04:6053951..6055765 (- strand)
	Solyc04g015740.3.1	transmembrane protein, putative	SL4.0ch04:6059620..6064860 (+ strand)
	Solyc04g015750.3.1	CobN/magnesium chelatase	SL4.0ch04:6065261..6071195 (- strand)
	Solyc04g015760.4.1	Protein N-terminal glutamine amidohydrolase	SL4.0ch04:6077102..6082474 (- strand)
	Solyc04g015770.1.1	Protein N-terminal glutamine amidohydrolase	SL4.0ch04:6098769..6099434 (- strand)
	Solyc04g015780.1.1	Protein N-terminal glutamine	SL4.0ch04:6101307..6101861 (- strand)
QTL4	Solyc08g074913.1.1	aspartic proteinase CDR1-like	SL4.0ch08:57171187..57172624 (- strand)
	Solyc08g074917.1.1	Eukaryotic aspartyl protease family protein	SL4.0ch08:57181223..57184722 (- strand)
	Solyc08g074950.2.1	Pectin acetylesterase	SL4.0ch08:57192306..57194206 (- strand)
	Solyc08g074955.1.1	Retrovirus-related Pol polyprotein from transposon TNT 1-94	SL4.0ch08:57198793..57199961 (- strand)
	Solyc08g074960.3.1	Major facilitator superfamily protein	SL4.0ch08:57211488..57215525 (+ strand)

Solyc08g074970.1.1	UDP-Glycosyltransferase superfamily protein	SL4.0ch08:57216128..57217573 (- strand)
Solyc08g074980.4.1	Receptor-like serine/threonine-protein kinase	SL4.0ch08:57220024..57223006 (- strand)
Solyc08g074990.3.1	Pectin acetylesterase	SL4.0ch08:57241396..57243647 (+ strand)
Solyc08g075010.4.1	DNAse I-like superfamily protein	SL4.0ch08:57258645..57269780 (- strand)

[a]Physical location based on the SGN SL4.0 assemb
