# Supplementary material for: Germplasm Screening Using DNA Markers and Genome-Wide Association Study for the Identification of Powdery Mildew Resistance Loci in Tomato
Source: Int J Mol Sci. 2022 Nov 6;23(21):13610. doi: 10.3390/ijms232113610 (PMC9657208; doi:10.3390/ijms232113610)
Supplement: Supplementary file 1 [file ijms-23-13610-s001.zip › Table S7.pdf]

Table S7. The genotyping results reconfirmed by converting chip single nucleotide polymorphisms (SNPs) AX-95809404 and AX-95781958 into cleaved amplified polymorphic sequence (CAPS) markers to 290 accessions used in the genome-wide association study (GWAS).

Phenotype	Accession	PDI (%)	SNPs	
			AX-95781958	AX-95809404
R	KNU17	0.00	T	A
R	A1161	0.00	T	A
R	A1162	0.00	T	A
R	A1216	0.00	C	G
R	LA1963	0.00	T	A
R	LA2932	0.00	T	A
R	LA2663	0.00	T	A
R	LA1274	0.00	T	A
R	LA1141	0.00	T	G
R	PI126445	0.00	T	A
R	LA1272	0.00	T	A
R	LA1674	0.00	T	A
R	LA1809	0.00	T	A
R	LA2744	0.00	T	A
R	LA2181	0.00	T	G
R	KNU16	4.76	T	A
R	LA0716	4.76	T	A
R	LA1340	5.56	T	A
R	LA2184	5.56	T	G
R	PI308182	6.67	T	A
R	PI134418	8.33	T	A
R	A1164	9.52	T	A
MR	A1155	11.11	C	G
MR	A1168	11.11	Y	R

MR	LA2852	11.11	C	G
MR	A1189	12.50	T	A
MR	A1170	13.33	C	G
MR	LA0722	13.33	T	A
MR	LA1461	14.29	T	A
MR	PI180721	14.81	T	A
MR	A1165	16.67	C	G
MR	A1169	16.67	C	G
MR	LA0407	16.67	T	A
MR	LA1777	16.67	T	A
MR	PI134417	16.67	T	A
MR	LA2312	19.05	C	G
MR	A1156	19.05	T	A
MR	LA1457	20.00	T	A
MR	A1151	20.00	C	G
MR	LA2185	20.00	T	A
MR	BT0911	20.83	C	G
MR	KNU11	22.22	T	A
MR	PI205037	22.22	C	G
MR	A1167	22.22	C	G
MR	A1173	22.22	C	G
MR	A1206	22.22	C	G
MR	A1208	22.22	C	G
MR	A1217	22.22	C	G
MR	LA1582	22.22	T	A
MR	A1236	23.33	C	G
MR	AVTO98039	25.00	T	A
MR	A1248	25.00	C	G
MR	A1234	25.93	C	G

MR	A1260	26.67	T	A
MR	LA1455	27.78	T	A
MR	A1198	27.78	C	G
MR	LA2779	27.78	T	A
MR	LA1246	27.78	T	A
MR	A1222	28.57	C	G
MR	LA4245	29.17	C	G
MR	A1163	29.17	T	A
MR	A1219	29.17	C	G
MR	A1227	29.17	C	G
MR	A1246	29.17	C	G
MR	A1247	29.17	C	G
MR	LA1028	29.17	T	A
MR	LA1941	29.17	T	A
MR	LA1578	29.17	T	A
MR	A1215	29.63	C	G
MR	LA1512	33.33	T	A
MR	LA2626	33.33	C	G
MR	LA2308	33.33	T	A
MR	BT0902	33.33	T	A
MR	BT0912	33.33	C	G
MR	PI254655	33.33	T	A
MR	A1157	33.33	C	G
MR	A1199	33.33	T	A
MR	A1223	33.33	C	G
MR	A1237	33.33	C	G
MR	A1243	33.33	T	A
MR	LA1406	33.33	T	G
MR	LA1930	33.33	T	A

MR	LA1946	33.33	T	A
MR	LA2533	33.33	Y	R
MR	A1207	36.67	C	G
MR	A1245	36.67	C	G
MR	A1244	37.04	C	G
MR	A1254	37.50	C	G
MR	LA1912	37.50	T	A
MR	LA3875	38.10	T	A
MR	A1187	38.10	C	G
MR	A1195	38.10	C	G
MR	A1218	38.10	C	G
MR	LA1208	38.89	C	G
MR	LA2845	38.89	C	G
MR	A1270	38.89	C	G
MR	LA0172	40.00	T	A
MR	A1160	40.00	C	G
MR	A1261	40.00	T	A
S	A1258	40.74	C	G
S	LA2561	40.74	T	A
S	A1205	41.67	C	G
S	A1226	41.67	C	G
S	LA1338	42.86	T	A
S	BT0909	42.86	T	A
S	LA1228	42.86	T	A
S	A1191	42.86	C	G
S	LA1701	44.44	C	G
S	LA4239	44.44	C	G
S	PI128216	44.44	T	A
S	LA1732	44.44	T	A

S	LA1668	45.83	T	A
S	A1238	45.83	C	G
S	Walter	46.67	C	G
S	A1152	46.67	C	G
S	A1211	46.67	C	G
S	A1225	46.67	C	G
S	A1159	47.62	C	G
S	A1283	48.15	C	G
S	LA2633	50.00	T	A
S	Floradel	50.00	C	G
S	LA4230	50.00	C	G
S	A1175	50.00	C	G
S	A1196	50.00	C	G
S	A1209	50.00	C	G
S	A1228	50.00	C	G
S	PI126925	50.00	T	A
S	SAL1875	50.00	T	A
S	LA4024	51.85	C	G
S	A1193	51.85	C	G
S	A1289	51.85	C	G
S	LA4247	52.38	C	G
S	LA1712	54.17	C	A
S	A1212	54.17	C	G
S	LA1464	55.56	C	G
S	LA4244	55.56	C	G
S	A1180	55.56	C	G
S	A1287	55.56	C	G
S	A1354	55.56	C	G
S	LA0446	55.56	T	A

S	A1262	56.67	C	G
S	A1315	56.67	C	G
S	LA1542	57.14	T	A
S	LA3136	57.14	T	A
S	A1172	57.14	C	G
S	LA4271	58.33	C	G
S	LA4279	58.33	C	G
S	A1263	58.33	C	G
S	A1312	58.33	C	G
S	A1356	58.33	C	G
S	A1255	60.00	C	G
S	A1314	60.00	T	A
S	A1259	61.11	C	G
S	LA1589	61.11	T	A
S	LA1621	61.90	T	A
S	LA3043	61.90	C	G
S	A1231	61.90	C	G
S	A1269	61.90	T	A
S	LA0373	61.90	Y	R
S	LA1545	62.96	T	A
S	A1158	62.96	C	G
S	A1233	62.96	C	G
S	A1252	62.96	C	G
S	A1281	62.96	C	G
S	LA1569	66.67	T	A
S	LA1623	66.67	T	A
S	LA1456	66.67	T	A
S	LA3882	66.67	C	G
S	A1224	66.67	C	G

S	A1232	66.67	C	G
S	A1257	66.67	C	G
S	A1275	66.67	C	G
S	A1349	66.67	C	G
S	PI126434	66.67	T	A
S	LA3892	70.00	C	G
S	LA1654	70.37	T	R
S	LA3878	70.37	C	G
S	A1256	70.37	C	G
S	A1355	70.37	C	G
S	A1230	71.43	C	G
S	A1203	72.22	C	G
S	A1229	72.22	C	G
S	A1353	72.22	C	G
S	A1276	73.33	C	G
S	LA2126A	74.07	C	G
S	King Humbert#1	75.00	T	A
S	PI296363	75.00	C	G
S	LA3999	75.00	C	G
S	Trujillo	76.67	C	G
S	LA1549	77.78	T	A
S	LA1632	77.78	T	A
S	2K6-8040	77.78	C	G
S	A1357	77.78	C	G
S	A1240	80.00	C	G
S	A1333	80.00	C	G
S	LA1346	80.00	T	A
S	LA3918	80.95	C	G
S	LA3928	80.95	C	R

S	LA3997	80.95	C	G
S	LA4264	81.48	C	G
S	A1241	81.48	C	G
S	A1308	81.48	C	G
S	A1267	83.33	T	A
S	A1313	83.33	C	G
S	LA1283	83.33	T	A
S	A1296	85.19	C	G
S	LA2664	85.71	C	G
S	LA1953	86.67	T	A
S	A1336	86.67	C	G
S	LA3975	86.67	C	G
S	LA2137	87.50	C	G
S	A1264	87.50	C	G
S	A1316	87.50	C	G
S	LA3869	88.89	C	G
S	A1210	88.89	C	G
S	A1265	88.89	C	G
S	A1280	88.89	C	G
S	A1350	90.00	C	G
S	LA3047	90.48	C	G
S	LA2131	91.67	C	G
S	LA2135	91.67	C	G
S	LA3267	91.67	C	G
S	A1250	91.67	C	G
S	A1348	91.67	C	G
S	A1351	91.67	C	G
S	LA3920	91.67	C	G
S	LA3960	91.67	C	G

S	LA3051	92.59	C	G
S	A1291	92.59	C	G
S	A1317	92.59	C	G
S	A1337	92.59	C	G
S	A1338	92.59	C	G
S	A1339	92.59	C	G
S	LA3866	93.33	C	G
S	A1201	93.33	C	G
S	A1268	93.33	T	A
S	A1341	93.33	C	G
S	A1342	93.33	T	A
S	LA2076	94.44	C	G
S	2K6-8201	94.44	C	G
S	LA3925	94.44	C	G
S	A1200	95.24	C	G
S	A1321	95.24	C	G
S	LA2163	95.24	T	A
S	A1327	95.83	C	G
S	LA3045	96.30	C	G
S	A1266	96.30	C	G
S	A1299	96.30	C	G
S	LA1547	96.30	T	R
S	A1251	96.67	C	G
S	A1293	96.67	C	G
S	A1328	96.67	C	G
S	A1340	96.67	C	G
S	LA2077	100.00	C	G
S	LA2078	100.00	T	A
S	LA3137	100.00	C	G

S	Fla.8233	100.00	C	G
S	LA3246	100.00	C	G
S	LA3344	100.00	C	G
S	LA3345	100.00	C	G
S	LA3867	100.00	C	G
S	LA3871	100.00	C	G
S	LA3874	100.00	C	G
S	LA3879	100.00	C	G
S	LA4246	100.00	C	G
S	LA4266	100.00	C	G
S	A1202	100.00	C	G
S	A1204	100.00	C	G
S	A1285	100.00	C	G
S	A1295	100.00	C	G
S	A1297	100.00	C	G
S	A1298	100.00	C	G
S	A1300	100.00	C	G
S	A1309	100.00	C	G
S	A1311	100.00	C	G
S	A1319	100.00	C	G
S	A1320	100.00	C	G
S	A1322	100.00	C	G
S	A1325	100.00	C	G
S	A1326	100.00	C	G
S	A1329	100.00	C	G
S	A1330	100.00	C	G
S	A1331	100.00	C	G
S	A1334	100.00	C	G
S	A1343	100.00	C	G

S	A1344	100.00	C	G
S	A1345	100.00	C	G
S	A1346	100.00	C	G
S	A1347	100.00	C	G
S	MM	100.00	C	G
