# Supplementary material for: Germplasm Screening Using DNA Markers and Genome-Wide Association Study for the Identification of Powdery Mildew Resistance Loci in Tomato
Source: Int J Mol Sci. 2022 Nov 6;23(21):13610. doi: 10.3390/ijms232113610 (PMC9657208; doi:10.3390/ijms232113610)
Supplement: Supplementary file 1 [file ijms-23-13610-s001.zip › Table S8.pdf]

Table S8. Tomato reference genome assembly information used for similarity analysis with SNP chip probe sequence.

| Species type | Species name | Assembly name | NCBI GenBank accession |
|---|---|---|---|
| Cultivated species | *S. lycopersicum* | SL2.40 | GCA_000188115.1 |
| Wild species | *S. chilense* | ASM601370v1 | GCA_006013705.1 |
| | *S. habrochaites* | Sohab10 | GCA_000577655.1 |
| | *S. pennellii* | SPENNV200 | GCF_001406875.1 |
| | *S. pimpinellifolium* | ASM1496433v1 | GCA_014964335.1 |
| | *S. arcanum* | Sorac 10 | GCA_000612985.1 |
